# Multinational strategy, institutions and spillovers: the role of institutions in knowledge spillovers in emerging markets

**DOI:** 10.1007/s10961-025-10230-w

**Published:** 2025-05-31

**Authors:** Sumon Bhaumik, Nigel Driffield, Katiuscia Lavoratori, Meng Song, Priit Vahter

**Affiliations:** 1 University of Sheffield, Sheffield, UK; 2 University of Warwick, Coventry, UK; 3 University of Reading, Reading, UK; 4 University of Leeds, Leeds, UK; 5 University of Tartu, Tartu, Estonia

**Keywords:** Inward FDI, Spillovers, Knowledge transfer, Institutions, Emerging markets, Random parameter model, F23, D22, R58

## Abstract

This paper delves into the pivotal role of institutions in facilitating knowledge spillovers, particularly in the context of multinational enterprises (MNEs) and knowledge transfer. While extant literature highlights the positive correlation between good institutional quality and knowledge transfer, this paper introduces a nuanced perspective. It argues for an inverted U-shaped relationship, suggesting that MNEs may exhibit reluctance in knowledge transfer to subsidiaries not only in environments with low institutional quality, as conventionally discussed, but also in those characterized by high institutional quality. In fact, there are beneficial implications of good institutions for domestic firms as well, enabling greater capacity to absorb knowledge and thereby emerge as competitors of MNEs. The paper demonstrates an inverted U-shaped relationship between spillovers, to capture knowledge transfer, and the quality of local institutions. This study provides valuable insights into the complex dynamics of knowledge spillovers, emphasizing the multifaceted influence of institutional environments on knowledge dissemination and economic development.

**Supplementary Information:**

The online version contains supplementary material available at 10.1007/s10961-025-10230-w.

## Introduction

Multinational enterprises (MNEs) are vital conduits for spillovers to domestic firms in host economies by facilitating the transfer of knowledge, technologies and best practices. This transfer can lead to significant productivity gains and innovation within the local economy, particularly in emerging markets, thereby boosting economic development. These beneficial exchanges occur through various mechanisms: as competitors, MNEs stimulate efficiency and innovation; as customers, they set higher performance and quality standards; as suppliers, they provide advanced inputs and foster learning opportunities; and as employers, they spread intangible knowledge through the mobility of trained workers from the MNE to the local labor market (Crone & Roper, 2001; Görg & Strobl, 2005; Zanfei, 2012).

The role of formal institutions in fostering knowledge spillovers has garnered significant attention in the international business (IB) and economics literature.^1^ There is a particular focus on the positive association between the quality of institutions and knowledge transfer from MNEs (Bhaumik et al., 2019). Well-developed formal institutions, which reduce transaction costs and safeguard intellectual property rights (IPR), are perceived as catalysts for the diffusion of knowledge across borders. However, while existing IB theory acknowledges the importance of good institutions in enhancing knowledge transfer, it tends to overlook their broader implications for MNE strategies and, by extension, for spillover benefits for domestic firms. This paper explicitly considers the strategic considerations of MNEs that go beyond the reduction of transaction costs in host countries and posit that well-developed institutions may actually limit the extent of foreign direct investment (FDI) spillovers.

We contend that strong institutions not only benefit MNEs but also empower domestic firms, enabling them to better absorb knowledge and technologies, and emerge as global competitors. Evidence suggests a positive association between institutional quality and a firm’s ability to allocate resources to productive activities and foster human capital accumulation (Vu, 2022), including innovative resources to improve innovation (Blanco & Goel, 2023). Building on this argument, literature shows that corruption deters FDI, but MNEs’ experience in dealing with corruption mitigates this (Dedho et al., 2025). However, from the local economy’s perspective, several issues are under-explored. For example, what is the nature of the experience that drives subsequent decisions, and how does this manifest itself after the investment? Specifically, does a firm’s experience facilitate better engagement with the local economy or inform its decision to isolate itself from uncertain local environments? This influences the nature of the knowledge transfer resulting from the investment. Together with the technological advancement of domestic supply chains and greater availability of skilled labor, this is likely to improve the technical efficiency and productivity of domestic firms. There is also evidence that institutional quality influences firm entry (Bruno et al., 2013), facilitating the Schumpeterian process essential for the efficient use of resources in a market economy. In other words, while a high level of institutional quality encourages knowledge transfer by MNEs, it also creates an environment where local firms can absorb and refashion this knowledge better and emerge as local and global competitors of the MNEs. Well-developed domestic institutions can provide local firms with an institutional competitive advantage, making them competitive both domestically and globally.

This nuanced understanding leads us to posit that MNEs may exhibit reluctance in transferring knowledge or technologies to subsidiaries not only in environments characterized by low institutional quality, as widely discussed, but also in those marked by exceptionally high institutional quality. Our paper challenges the conventional wisdom around the role of institutions in facilitating knowledge transfer, which envisages a linear positive relationship between institutional quality and knowledge transfer (and spillovers), by suggesting an inverted U-shaped relationship between institutional quality and knowledge transfer. In other words, from a host country perspective, there may exist an optimal level of institutional quality if it wants to maximize FDI spillovers.

The contributions of this paper center around a better understanding of the interactions between institutions, the absorptive capacity of domestic firms, and international knowledge transfer by MNEs. From a theoretical standpoint, it extends the literature on the implications of host country institutions on MNE strategy beyond discussions about transaction costs, learning and institutional arbitrage towards a trade-off between transaction cost and the threat of credible competition. While other papers have considered the strategic considerations of MNEs in the context of technology transfer (Glass & Saggi, 2002), and while the literature acknowledges concerns about domestic firms’ absorptive capacity that, in part, is dependent on local institutional quality, this is the first paper to systematically explore the relationship between host country institutional quality and MNE strategy in knowledge transfers and spillovers. This focus on institutions is important for two reasons. Firstly, the current absorptive capacity of domestic firms is partly a function of past institutional quality. Secondly, current institutional quality informs MNEs’ expectations about the nature of both absorptive capacity and the ability to manage their technology in the home country.

Further, by introducing this trade-off into the discussion around knowledge transfer, we provide a theoretical basis for explaining the variation in reported spillover effects documented in the literature. Building on this, we develop a deeper understanding of what has often been referred to as the “missing part of the jigsaw” in spillover analysis, which is the distinction between the incentive for a foreign firm to become embedded in its host region and the incentive to prevent knowledge leakage that would hurt its competitive advantage globally (Santangelo et al., 2019). We also contribute to the discourse on knowledge transfer for productivity and economic growth by exploring potential mechanisms and sources for such transfer (Acs et al., 2013; Audretsch & Belitski, 2024). Our focus is on the presence of foreign MNEs through FDI in the local economy (Driffield et al., 2010; Castellani et al., 2024), an area underexplored in the entrepreneurship literature (Laursen et al., 2006; Amoroso et al., 2018), with a few exceptions (Andersson et al., 2022).

We argue that when considering whether to integrate into the host economy, MNEs face a trade-off between institutional quality and the absorptive capacity of local firms when determining their scale and scope of embeddedness in the host economy. We demonstrate that considering firm strategy, and how institutions and absorptive capacity influence MNEs’ strategies, better explains the variation in reported spillover effects. Our narrative also poses an important question for host country policymakers, where there is significant headroom for improvement in institutional quality. While improving institutional quality may be worthwhile, for countries prioritizing knowledge transfer by MNEs and spillover effects, our framework suggests that there may be an optimal level of institutional quality beyond which net economic benefits may be insignificant. In the IB and economics literature, this adds complexity to the policy choices of host country governments and their bargaining with MNEs (Bhaumik et al. 2024), by introducing the possibility that secular improvements in institutional quality may not always be in the interest if they aim to maximize spillover benefits.

Since knowledge transfer is difficult to observe directly, we use spillover from specific MNE projects as a proxy. Our key hypothesis is that there is an inverted-U relationship between local institutional quality and observed spillover effects. We empirically test this with a unique dataset of about 1,300 investment projects by 621 MNEs from developed countries in the automotive and computer industries, from Moody’s Orbis-Cross Border Investment (CBI) database. These projects take place in 201 host cities in 30 emerging economy destinations. Using Moody’s Orbis database, we collect financial data for the local companies in these cities and compute their labor productivity. We estimate the effect of each FDI project on local productivity and investigate why this effect varies across projects and locations using a random parameter model. Our empirical analysis supports the proposed inverted U-shaped relationship.

The paper proceeds as follows: Sect. 2 discusses the literature on MNE strategy, institutions and knowledge spillovers, and develops the hypotheses. Section 3 outlines the empirical strategy. Section 4 presents data and measures. Section 5 discusses the main findings, and Sect. 6 concludes.

## Theoretical framework and hypothesis development

### Absorptive capacity and spillovers

Absorptive capacity, typically measured by the productivity of domestic firms and sectors, is an important determinant of spillovers in the host country (Bournakis et al., 2022; Castellani et al., 2024). Firms with high absorptive capacity can derive greater value from joint patenting with foreign multinationals (Mathew et al., 2024). They can also integrate FDI spillovers with internal knowledge to identify new business opportunities and drive product innovation (Zhao et al., 2019; Guo et al., 2022). Countries more connected with global FDI networks experience greater technological advancement, particularly if they possess higher absorptive capacity (Sultana & Turkina, 2020). For less developed countries, absorptive capacity is important for transforming FDI knowledge spillovers into innovation, thereby reducing in-house R&D expenditure (Duan et al., 2021). Dunning’s (1986) investment development cycle illustrates how inward investment and accompanying knowledge transfer can help countries upgrade their technology and development levels. This is found in several Asian countries, notably Korea (Buckley et al., 2022), Malaysia (Noor et al., 2002) and China (Wang & Kafouros, 2020).

The concept of absorptive capacity can be extended to regions and cities (Roper & Love, 2006; Criscuolo & Narula, 2008; Caragliu & Nijkamp, 2012, 2016). An accumulated stock of cognitive capital enables these entities to identify, absorb and utilize external knowledge effectively. Studies at the regional level in Europe show that lower regional absorptive capacity hampers the ability to decode and exploit new knowledge, whether locally produced or sourced externally (Caragliu & Nijkamp, 2012). Conversely, higher regional absorptive capacity attracts knowledge spillovers even when other regions invest in R&D (Caragliu & Nijkamp, 2016). Several studies have found that the effect of inward FDI on innovation and production is positively moderated by regional absorptive capacity. For example, in Chinese regions, Fu (2008) finds that the impact of inward FDI on regional innovation capacity depends on the availability of absorptive capacity and innovation-complementary assets. Regions hosting most of China’s R&D activities, top universities, and research institutes, with a pool of highly educated and skilled workers, have seen significant promotion of regional innovation capacity due to FDI. Similarly, Smith and Thomas (2017) show that Russian regions with higher absorptive capacity, measured by human capital levels, benefit from FDI-related technological spillovers.

However, this literature typically focuses on the ability of host country firms to assimilate knowledge, rather than the relationships between this capacity and the MNEs’ decisions to engage in knowledge transfer between parents and their affiliates, and their integration into the host economy. Zahra and George (2002) propose that a regime of appropriability affects firms’ ability to protect the advantages of new products or processes. When appropriability is low and knowledge spillovers are prevalent, firms are less likely to invest in developing absorptive capacities. Conversely, when appropriability is strong, firms are more likely to develop absorptive capacities and create competitive advantages due to the high costs associated with imitation. Technology-leading MNEs tend to engage in fewer alliances and exchange fewer workers with domestic firms in host countries to avoid inadvertent knowledge spillovers to local firms. Hence, host countries might benefit more from lower-ranking MNEs than from technology leaders (Crescenzi et al., 2022).

The wider literature also acknowledges that MNEs are concerned about the absorptive capacity of domestic firms. Alcácer and Chung (2007) find that FDI in the U.S. considers both the potential gains from knowledge spillovers from host countries and the risks of inadvertent spillovers to local competitors. Less technologically advanced FDI can absorb more appropriable knowledge produced by local industrial innovation activities and tends to favor such locations in a host country. In contrast, more technologically advanced FDI tends to avoid locations with high levels of industrial activities to protect their knowledge, preferring locations with high levels of academic innovative activities that produce more basic and less appropriable knowledge. This approach helps such FDI gain knowledge spillovers in a host country, while protecting their knowledge from local competitors. Crescenzi et al. (2022) observe that technology-leading MNEs tend to invest in less developed regions with low absorptive capacity to protect their knowledge from spilling over to competitors.

This argument highlights the complexity of the relationship between spillovers and absorptive capacity. MNEs require a certain level of absorptive capacity in the host country to source certain inputs and develop local supply chains. Building on Girma (2005), a given level of absorptive capacity is necessary for local firms to assimilate knowledge transfer from inward investors. However, as absorptive capacity increases, the knowledge gap between inward investors and local firms decreases, leading to diminishing benefits from spillovers. As absorptive capacity increases, the distance between the local firms and the technology frontier declines, reducing the scope for further learning effects.

### Institutions and knowledge transfer

The traditional spillovers literature (for review papers see Meyer & Sinani, 2009; Perri & Peruffo, 2016; Keller, 2021) often adopts an augmentation of Dunning’s (1979) OLI framework, focusing on the incentives for MNEs to engage in knowledge transfer to exploit their ownership advantages in new markets. However, this approach overlooks the crucial role of country-specific advantages, particularly institutional quality, in explaining variations in knowledge transfer and spillovers.

Knowledge transfer can occur at the time of the investment, with technology or knowledge embedded in physical capital or accompanying the initial investment in the form of managerial skills. It can also occur subsequently as part of affiliates’ development. Specifically, knowledge transfer by MNEs increases with the quality of host country institutions (Tihanyi & Roath, 2002). MNEs seek to protect their ownership advantage, which requires robust intellectual property protection (Javorcik, 2004; Branstetter et al., 2006).^2^

Weak property rights protection hampers inter-firm collaboration beyond the uncertainty of appropriation (Buckley et al., 2020). MNEs are unlikely to invest in physical capital in a host country without adequate property rights protection. If the sanctity of property rights is challenged in court, MNEs would require the rule of law to prevail before transferring technology associated with their ownership advantages. As such, good institutions can foster inter-organizational trust necessary for knowledge transfer (Garcia-Vega & Huergo, 2017), based on the strength of Coasian institutions. Finally, quality institutions can help MNEs overcome weaknesses in their international experience and liability of foreignness (Putzhammer et al., 2018). Good host country institutions can reduce the transaction costs of acquiring local assets, including contractual relationships with local firms that can be part of their supply chains, making the business venture profitable (Hennart, 2009).

Institutional quality is not static and can evolve over time. Countries often adopt market-friendly institutions favoured by international and supranational organizations. The evolution of Coasian institutions in Central and Eastern Europe post-socialism and the adoption of market-friendly institutions in developing countries are examples of this process. However, transplanted formal institutions may not work as intended if the local context is not predisposed towards them (Berkowitz et al., 2015). The political economy literature suggests that institutions such as property rights protection and the rule of law result from the political process within a country, which determines the limits of expropriation by a predatory state (Besley & Ghatak, 2010) and the interests of the players demanding better formal institutions (Hoff & Stiglitz, 2004). Recent IB literature suggests that MNEs adopt strategies to survive and shape relevant institutions in specific host country contexts (Regner & Edman, 2014). However, for any given country, the quality of these institutions is often stable over time unless there is significant external intervention, as seen in Central and Eastern European countries in the 1990s. Therefore, it is often possible to focus on cross-sectional differences in institutional quality.

To summarize, while institutions can evolve over time, the importance of well-developed institutions for deeper MNEs’ engagement with a host country and subsequent knowledge transfer is well established in the literature. Strategies around co-developing new technologies by MNEs in host countries are influenced by host country institutions such as intellectual property rights (Nandakumar & Srikanth, 2016). Recent literature highlights the possibility of “opportune enforcement” of IPRs (Prud’homme & Tong, 2024), which has implications for the institutional quality and knowledge transfer relationship. However, the broad emphasis on institutional quality, particularly property rights, remains the cornerstone of the literature on technology transfer by MNEs.

### Institutions and spillovers

The above discussion suggests that strong formal institutions facilitate greater spillovers, in part by fostering greater absorptive capacity on the domestic sector, and in part by way offering greater IPR protection for knowledge that is transferred from parent to affiliate. The literature widely acknowledges institutional quality’s role in knowledge spillovers. Villar et al. (2020) contrast spillovers in advanced and emerging economies, emphasizing institutional quality in export spillovers. Dogan and Wong (2020) find that institutional quality enhances FDI spillovers in a set of ASEAN countries but do not detail the mechanisms. Similarly, Nam et al. (2023) and Lebedev and Peng (2024) highlight local institutions’ importance in spillovers, but do not explore the nature of this relationship in detail. More recently, Slesman et al. (2021) show that a threshold level of institutional quality is needed for inward investment to foster local entrepreneurship.

High-quality institutions further enhance spillovers by improving labor market efficiency (Nickell and Layard, 1999), enabling skilled worker mobility, which in turn fosters knowledge spillovers via labor turnover. Fair competition laws create a level playing field, driving domestic firms to innovate under competitive pressure (Fabrizio et al., 2017). Additionally, strong institutions facilitate the establishment of backward and forward linkages between domestic and foreign firms by ensuring reliable infrastructure, utilities, and business support services (Schøtt & Jensen, 2016). As a result, domestic firms can derive greater benefits from these linkages with MNEs, allowing domestic firms to benefit more from MNE linkages.

However, an important distinction has to be made about the role of institutions in influencing the absorptive capacity of local firms and MNE strategies. We posit that the absorptive capacity of domestic firms in period *t* is influenced by the quality of institutions in periods *t-i*, when *i* = 1, 2, 3, …., i.e., observed absorptive capacity at any given time is a cumulative outcome of operating in a certain institutional context over a period of time. However, given the forward-looking nature of strategy, current institutional quality shapes the expectations of MNEs about the future quality of local institutions and the implications of local institutional quality on future outcomes. These expectations, in turn, influence its strategy, in particular, about knowledge transfer. Accordingly, we discuss next an outcome of good institutions on economic outcomes that matter for MNE strategies, specifically, on the capabilities of domestic firms.

### Institutions and domestic firm capabilities

In contexts where institutions do evolve and improve in quality over time, market efficiency in capital, labor and intermediate goods markets is improved (Cuervo-Cazurra & Dau 2009). Thus, local firms are better able to take advantage of the opportunities that inward investors create. Their ability to access capital markets to invest in key resources and undertake strategic repositioning/restructuring is enhanced by more secure property rights as well as by improvement in shareholders’ and creditors’ rights. Similarly, better surety concerning enforcement of contracts enables firms to grow and generate scale economies (Van Biesebroeck, 2005), both because it provides better access to external capital and also because it enables firms (or entrepreneurs) to focus on productive activities rather than activities such as lawsuits (Sobel, 2008; Giacomelli & Menon, 2017; Lopez-Martin & Perez-Reyna, 2021). Better contract enforcement also enables firms to use complex and customized intermediate goods (Ma et al., 2010), thereby enabling them to move up the value chain. Finally, these developments also facilitate entrepreneurial activity and Schumpeterian churn (Aidis et al., 2009; Baumohl et al., 2019; Audretsch & Fiedler, 2022), by reducing barriers to entry (and exit) that ensures the survival of the most productive or efficient firms (Holmes & Schmitz, 2010; Backus, 2020). In sum, the competitiveness of emerging market firms grows with improvement in the quality of their formal institutions.

This process can perhaps be best understood by focusing on the market for financial resources, which is key to business investment as well as market entry by new firms. Efficient financial markets provide domestic firms with the resources to innovate and adapt technologies (Aggarwal & Goodell, 2010). In emerging market contexts, financial markets do not operate well because of institutional voids. For example, weak property rights protection can make it difficult to post collateral, which is necessary to overcome potential adverse selection problems (Bester, 1987; Jimenez et al., 2006; Godlewski & Weill, 2011),^3^ thereby making it difficult for firms to raise external capital (Maurer & Sharma, 2001; Kerekes & Williamson, 2008). Greater access to outside capital is also facilitated by improvements in the quality of contract enforcement (Quintin, 2008), which is another pillar of formal institutions.

Stronger institutions, such as improved education systems, infrastructure, and regulatory frameworks, enhance domestic firms’ competitiveness: better institutions produce a more skilled workforce capable of absorbing advanced managerial and technological knowledge introduced by MNEs (Corradini et al., 2023).

Improvement in formal institutions can also facilitate the internationalization of these firms. For example, better-functioning capital markets and better contract enforcement can reduce the incentives for firms to adopt organizational forms that involve family control and business groups that reduce the likelihood of outward FDI for a variety of reasons (Bhaumik et al., 2010). To be sure, the existing literature suggests that the internationalization of emerging market firms may both be driven by a desire to escape weak institutions (Gaur et al., 2018; Cui & Xu, 2019) and also be facilitated by institutional support that these firms can leverage (Cuervo-Cazurra & Genc, 2008; Landau et al., 2016), but it is reasonable to surmise that weak home institutions raise the cost of internationalization overall.

Therefore, high-quality institutions are expected to be associated with greater absorptive capacity of local firms in the future. More importantly, it creates a virtuous cycle whereby this absorptive capacity and the ability to develop proprietary technology grows over time. As we discuss in the following section, this poses a strategic challenge for an MNE.

### Multinational strategy

The traditional IB discourse has focused largely on transaction costs associated with weak institutions in host country contexts, where MNE strategy revolves largely around the choice of ownership in the host subsidiary (Driffield et al., 2016) and around the choice of entry mode (Meyer et al., 2009), or the inability of local firms to absorb that knowledge (Girma, 2005; Castellani et al., 2024). This literature adopts a two-stage approach, where the first stage focuses on the investment decision in the context of variations in institutional quality (Dedho et al., 2025) and the second on the decisions regarding technology management and engagement with the local economy subsequent to the decision Amiti et al. (2024). More recent research has focused on MNE political strategies and, among other things, the ability of these organizations to influence the nature of formal institutions and policy choices of governments in host countries. Implicit in this literature is the desire of MNEs to protect their ownership advantage by way of reducing the loss of IPR, but, while the literature on spillovers has discussed the importance of host country institutions in shaping the absorptive capacity of local firms, as well as the concerns and strategies of MNEs around this absorptive capacity, the role of host country (or local) institutions in shaping MNE strategies has not been fully explored and we do that in the rest of this sub-section.

The traditional view of international knowledge transfer shows that the process initially requires knowledge transfer from the MNE to its foreign affiliate, followed by the potential for the generation of externalities (i.e., spillovers) from the foreign affiliates to domestic firms (Driffield et al., 2010). The extensive spillover literature concerns itself exclusively with the final stage, and this presents an identification problem (Driffield et al., 2024). In practice, there is no guarantee that either condition will be fulfilled, and while it is assumed that international technology transfer is a prerequisite for spillovers, the identification of this stage is seldom discussed within the spillover literature. Not all affiliates automatically have access to the leading technology and knowledge of their parent company, and, notwithstanding the possibility of inadvertent leakage, MNEs frequently go to considerable lengths to internalize their knowledge and prevent or control its transfer to third parties. Therefore, even if intra-firm knowledge transfer occurs, there is no guarantee that the domestic economy in which the affiliate is located will benefit as a result. This raises two questions, which we seek to address here. The first is that to understand the nature of these processes, particularly in the context of FDI to emerging markets, one must first understand the MNEs’ strategy regarding the decision to invest, and, in turn, one must understand the nature of the location in question. Therefore, we develop a framework by drawing on the literature concerning multinational strategy in emerging economies, and specifically the decisions that firms make regarding the degree of embeddedness, given local formal institutions. We pose the question, therefore, as to how an MNE would strategize about integration in an emerging market context, when it knows that improvement in local formal institutions not only protects it from expropriation and facilitates market transactions by reducing transaction costs, but this improvement in institutional quality also creates more productive and competitive domestic firms that can absorb frontier technologies more easily.

Building on Rugman and Collinson (2006), we argue that companies face a trade-off between integration to benefit from local supply chains and to foster relationships with local stakeholders, and retaining/defending the protection of their property rights. For simplicity, our model presumes three potential states of institutional quality, low, medium or high, and similar variation in absorptive capacity of host country firms. When institutional quality is *Low*, the threat of expropriation is high and the incentive of the MNE to integrate into the host country market is low. At the other extreme, if institutional quality is *High*, the likelihood of expropriation of the MNE’s “technology” is low and hence there is a greater incentive to integrate more with the host country. However, this advantage is offset by the possibility that if greater integration leads to greater spillovers, given the greater absorptive capacity and capabilities of the domestic firms, in the longer run the MNE may face a more competitive local (and perhaps even global) market. This follows from our discussion about institutional quality and the capabilities of local firms in the previous section. On balance, therefore, it is reasonable for the MNE to not pursue a high degree of integration into the host country market. In other words, the best response of the MNE might be to integrate less with the host country market when the host country’s institutional quality is both *High* and *Low*. This gives us the following propositions:

#### Proposition 1

*When the institutional quality in a host country is low/weak, a MNE is less likely to transfer technology to a host country because of the attendant risks associated with the loss of IPR and, by extension, its ownership advantage*.

#### Proposition 2

*When the institutional quality in a country is high/strong, a MNE is also less likely to transfer technology to a host country because of the risk of creating local and global competitors by way of spillovers that are strong when the absorptive capacity and capabilities of local firms are high*.

**Fig. 1:**
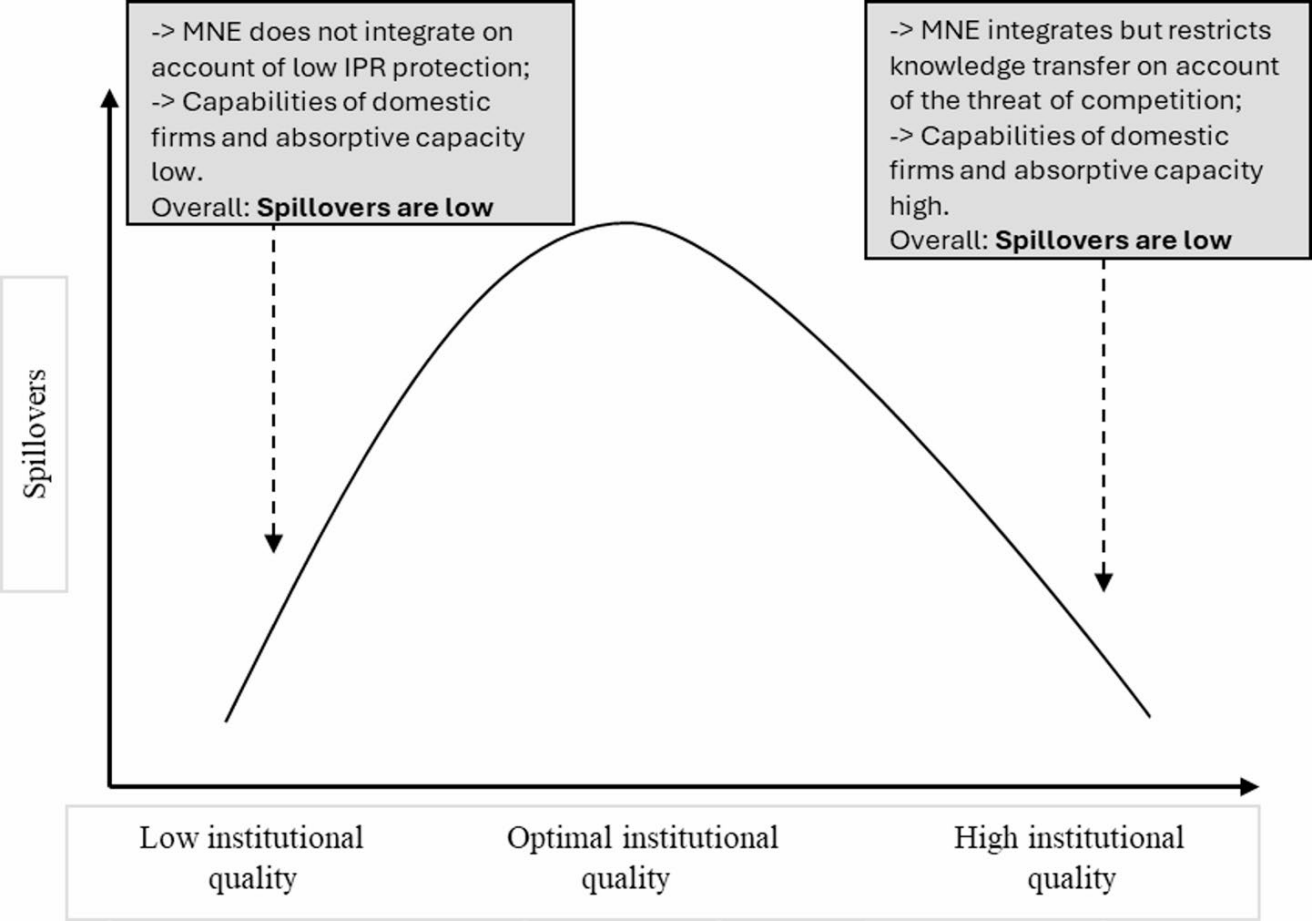
Conceptual scheme of institutions, knowledge transfer, and spillovers

Proposition 2 Highlights that beyond an optimal level of institutional quality, with further improvements in institutional quality, increased competition may lead to reduced knowledge transfer from MNEs to domestic sectors. This aligns with the inverted U-shaped relationship between institutional quality and spillovers, where better institutional quality initially enhances spillovers but can eventually lead to a strategic reduction in knowledge transfer by MNEs as domestic firms grow more competitive. Strong institutions facilitate knowledge spillovers by reducing transaction costs and improving contract enforcement; however, they also enable domestic firms to accumulate absorptive capacity and enhance their capabilities, creating a credible competitive threat to MNEs over time. This is captured in Fig. 1.

Our framework then allows us to develop two hypotheses that both emphasize the importance of absorptive capacity in explaining knowledge transfer and distinguish between the effects of absorptive capacity and institutional quality. Implicitly, our framework considers the incentive for a firm to engage in knowledge transfer from its home country to the host country, as well as the scale and scope of knowledge transfer from the affiliate to the local economy. We argue that to develop an empirical construct for this, it is necessary to unpick the respective roles of institutions and the nature of local firms. Therefore, we focus on the role of absorptive capacity at the level of the local firm, as well as the non-linear relationship between host country institutions and spillovers.

Based on the above discussion, we hypothesize that:

#### Hypothesis 1

*Given the level of knowledge transfer by MNEs, spillovers increase with the level of absorptive capacity, but at a decreasing rate*.

#### Hypothesis 2

*For a given level of absorptive capacity, the relationship between spillovers from FDI and institutional quality will exhibit an inverted U-shaped relationship*.

## Empirical strategy and methods

### Two-stage random parameter model

Our paper aims to investigate the role of the absorptive capacity of domestic firms and the quality of institutions of the host environment in shaping MNEs’ strategies when investing in emerging markets and, therefore the impact on the local economy. In doing so, our empirical strategy needs to allow us to investigate (1) the effect of inward FDI on productivity returns of local firms (i.e., spillovers), and (2) whether the size of such spillovers can be the result of host local characteristics influencing MNEs’ strategies in the host location, and in turn, whether different investment projects can generate heterogenous spillover returns based on the characteristics of the local context in which the investment takes place. Thus, we approach this investigation with a two-step econometric strategy exploiting some features of random parameter (mixed-effect) models (RPMs). In the first stage, we estimate the association between inward FDI projects and local productivity using a random parameter model. RPMs allow to control for unobserved heterogeneity in regression coefficients, as they allow coefficients to vary by group (e.g., firm, region, project), and directly model such heterogeneity. From this first stage, we derive an average coefficient (similar to standard regressions) and a standard deviation, signalling a heterogeneous influence of the foreign presence across groups (i.e., in our case, FDI projects). In the second step, we exploit one of the properties of RPMs and we predict group-level parameters. We use this predicted parameter as a dependent variable in a second-stage regression to investigate whether and how host country characteristics can explain its variation across investment projects (Greene et al., 2009; Hawk & Pacheco-de-Almeida, 2018; Castellani & Lavoratori, 2020).

#### First stage–effect of inward FDI on local productivity

Random parameter models represent an appealing response to go beyond average effects and explicitly model heterogeneity, something that standard regression models cannot do directly (Alcácer et al., 2018), as a special case of multilevel (or hierarchical) linear models, relevant when the data present a hierarchical structure. In our case, the data present a two-level structure, where the first level is represented by the local firms exposed to the presence of the focal MNE’s investment project, which represents the second (higher) level: local firms (observations) are nested within investment projects in a given city. This allows us to predict the heterogenous effect at the investment project level on the host local productivity. In other words, our units of observation are pairs of local firms (*j*) and focal investments (*i*) in a given city (*c*) where the investment project takes place. Considering a linear regression model formalized as follows,

1 $$\:{Y}_{ij}=\alpha\:+{\beta\:}_{}{X}_{1ic}+\theta\:{X}_{2c}+{\delta\:}_{}{{{X}_{3j}}_{}+\epsilon\:}_{ij}$$

where $\:{Y}_{ij}$ is the dependent variable, $\:{X}_{1ic},$ $\:{X}_{2c},$ and $\:{X}_{3j}\:$are a set of project-location, location and local firm characteristics, respectively. If we allow for differential intercepts at the project level, the randomness of the intercept is introduced ($\:\alpha\:$), whereas $\:{\beta\:}_{},$ $\:\theta\:$ and $\:{\delta\:}_{}\:$are fixed coefficients equal to all firms. The coefficient $\:\alpha\:$ can be now expressed as:

2 $$\:{\alpha\:}_{i}={\gamma\:}_{00}+{u}_{i0}$$

where $\:{\gamma\:}_{00}$ is the overall mean, and $\:{u}_{i0}$ is the random part of the model consisting of higher-level residuals as the distance from the sample mean related to the project-level group *i*.

So far, the model assumes homogeneous average effects associated with the explanatory variables, hiding possible heterogeneous patterns. As discussed in Sect. 3.1, RPMs can explicitly model such heterogeneity, allowing for the randomness not only in the intercept but also in the slope of some explanatory variables by estimating group-specific parameters. Thus, considering the variable $\:{X}_{1ic}$ as our variable of interest, in (1) it can be set as random at the project level *i*, and the model can be formally extended as follows,

3 $$\:{Y}_{ij}={\gamma\:}_{00}+{u}_{i0}+{\beta\:}_{i}{X}_{1ic}+\theta\:{X}_{2c}+{\delta\:}_{}{{{X}_{3j}}_{}+\epsilon\:}_{ij}$$

where

$$\:{\beta\:}_{i}{X}_{1ic}={\beta\:}_{0}{X}_{1ic}+{u}_{i}{X}_{1ic}\:\left(4\right)$$

namely $\:{\beta\:}_{0}\:$is the overall mean slope and $\:{u}_{i}$ is the slope deviation for project *i* for the variable $\:{X}_{1ic}$. $\:\beta\:$ is allowed to vary by project *i*, with a probability density function g($\:.$)^4^, decomposed in its mean coefficient ($\:{\beta\:}_{0}$) similar for all firms and a standard deviation ($\:\sigma\:$). In Eq. (4), $\:{u}_{i}$ is the deviation from the mean coefficient $\:\beta\:$ associated with project *i*, randomly distributed with mean zero and standard deviation $\:\sigma\:$. A significant $\:\sigma\:$ reveals that different projects may generate different benefits for the host local economy. Since each investment project is a one-time event, we treat the data as a cross-section analysis, and the explanatory variables are lagged by one period to address potential endogeneity problems. We also include year, industry and host country dummies to control for unobserved factors.

#### Second stage–heterogenous returns from inward FDI

There are two main traditional approaches to account for heterogeneity. The first one consists of splitting the sample into subsamples by some relevant characteristics under investigation, performing the analysis for each subsample and comparing coefficients; the second consists of using interaction terms between the variable of interest and firm/host location characteristics as an additional explanatory variable. However, these two approaches have several limitations (for a detailed discussion, see Alcácer et al., 2018), especially when several moderating factors operate simultaneously, or non-linear relationships exist (Lavoratori & Castellani, 2021; Castellani et al., 2024).

Based on the results from the first stage, if the variable of our interest will present a significant standard deviation of the random parameters, it means that a source of heterogeneity exists and we aim to understand which factors may explain it.

In so doing, we exploit one of the characteristics of RPM and we estimate project-level coefficients ($\:{\beta\:}_{i}$) by predicting the project-specific random component $\:{u}_{i}$, which captures the effect of our variable of interest ($\:{X}_{1ic}$), i.e. the capital expenditure of the focal investment *i* in the city *c*, on the local productivity, across investment projects. This vector can be used as a dependent variable in a second-stage regression, where several explanatory variables can enter the model simultaneously to explain its variation. Following Saxonhouse (1976) and Hornstein and Greene (2012), we use this predicted parameter $\:{\beta\:}_{i}$ as a dependent variable in a second-stage OLS estimation. More formally and following our hypotheses,

5 $$ \:Spillovers\:\left( {\beta \:_{i} } \right) = \gamma \:_{0}  + \:\gamma \:_{1} \:X\: + \:\gamma \:_{2} \:X^{2}  + \sum {\:_{{g = 3}}^{n} } \gamma \:_{g} X_{g}  +  \in  $$

where, $\:{\beta\:}_{i}\:$(*Spillover*) is the vector of predicted project-specific coefficients, $\:X$ represents our main explanatory variables and their quadratic form (i.e. quality of institutions and absorptive capacity), while $\:{X}_{g}$ are host city, industry and country controls. In the first stage, we also obtain the standard error of each estimate together with the coefficient of interest. These estimated standard errors are then used as weights for the second-stage regression to control for heteroscedasticity issues potentially affecting estimated parameters (Saxonhouse, 1976; Hornstein & Greene, 2012).

## Empirical setting

### Data

Given the aim of the paper and our empirical strategy, we organize the data collection in three steps. First, we collect data on FDI projects undertaken by companies from developed countries investing in developing countries based on the classification provided by UNCTAD^5^, and operating in four manufacturing sectors classified by Eurostat^6^ as medium-high and high-technology sectors where FDI spillovers are more likely to happen, that is the manufacturing of electrical equipment, computers, machinery and equipment and automotive^7^. Our analysis relies on data from the Orbis CBI database, which covers detailed information on greenfield investments and merger and acquisition (M&A) deals worldwide, compiled and made available by Moody’s (formerly by Bureau van Dijk). The CBI dataset provides detailed information on the investment projects, such as the parent company name and BVD identification number, its home country, the destination country and city of the project, the type of investment (greenfield vs. M&A deals), and the capital expenditure in USD. All projects missing data for any of these relevant variables were removed from our sample^8^. Thus, our final sample is composed of 1,266 investment projects over the period 2013–2017. 8% of these projects are M&A deals, but the majority (92%) are greenfield projects. The FDI projects are from 28 developed countries, more specifically, as reported in Table A.1 in the Appendix, around 18% of projects are from Asia (Japan), 45% from Europe and 36% from North America, where the USA and Germany represent the main investors. These investment projects take place in 30 developing countries in 201 host cities, while 10% of projects go to Mexico, the majority of investments take place in Asia, leaving 3% of projects in Africa. The distribution of projects by destination country and city is reported in Tables A.2 and A.3.

Second, from the first step, we have the list of host cities we are interested in as recipient locations of the FDI projects under investigation. We collect data on the local firms operating in these cities from Moody’s Orbis database over the period 2013–2018, and operating in the four main sectors, along with three additional sectors linked through backward and forward linkages, i.e. NACE Rev. 2 sectors 24-Manufacture of basic metals, 25-Manufacture of fabricated metal products, except machinery and equipment, 33-Repair and installation of machinery and equipment. This returns about 33,000 local firms, 65% of which operate in the four main sectors (NACE 26-27-28-29), and the remaining 35% in backward/forward sectors (NACE 24-25-33). The distribution of companies across cities is reported in Table A.4 in the Appendix. These companies and their productivity will be the basis of analysis in the first stage equation, where the unit of analysis is the dyad local firm-FDI project.

Finally, we collect relevant data at the host country level from several sources, such as the World Bank, World Economic Forum, and Center d’Etudes Prospectives et d’Informations Internationales (CEPII).

### Measures

#### First stage – Effect of inward FDI on local productivity

As discussed in Sect. 3.1.1, in the first stage we estimate the effect of FDI projects on the productivity of local firms located in the cities where these investments take place. We then use this information to compute a measure of spillovers, using the estimated coefficients.

*Dependent variable*. Our dependent variable is a measure of the productivity of local firms located in the selected cities, and we measure their labor productivity as the output (revenue) per employee.

*Independent variable*. The main explanatory variable is a measure of the foreign presence in the host city. Using information from CBI, we measure the foreign investment as the USD value of capital expenditure related to each focal investment project (*FDI project value*).

Since we can have multiple investors in the same city in the same year, we control for the presence of other foreign multinationals in two ways: first, by computing the sum of capital investment of other FDI projects in the city-year excluding the focal one (*Capital value other FDI projects*); second, by calculating the proportion of capital investment from other projects (excluding the focal one) to the total capital expenditure in the city-year (*Share of other FDI projects*).

We also include traditional controls of host firm and city characteristics. More specifically, we control for the age of the local company, and its size. Firms with more than 250 employees are classified as large, medium-sized with 50–250 employees and small with less than 50 employees.

We also compute the total assets of firms located in the host city (*Tot. assets in city-sector*), the number of companies in the city operating in the same sector of the firm (*No. firms in sector-city*) and the number of firms in the city operating in other sectors (*No. firms in other sectors-city*), as measures of (specialization and diversification) agglomeration economies. We also include industry, year and country fixed effects to account for unobserved aggregate level drivers of productivity. Explanatory variables are lagged by one year (t-1).

#### Second stage – Heterogenous returns from inward FDI

The second-stage regression aims to investigate the host country and project factors that can explain the heterogeneous returns on local productivity.

*Dependent variable: Spillovers.* Using the results from the first stage, we predict the “spillover parameter” for each FDI project, which measures the extent of spillovers from the presence of the focal MNE in the local economy. We use this parameter as the dependent variable in our second-stage regression.

*Independent variables*.

To test Hypothesis 1, we compute a proxy of *absorptive capacity* in the local economy as the current level of aggregate labor productivity at the host city. We follow Olley and Pakes (1996) and compute the *aggregate productivity* at the city level as a share-weighted average of the firm labor productivity of all firms gathered from Orbis operating in the selected sectors. In other words, we sum productivity levels using firm-level output shares as weights. We also compute its quadratic term to test possible non-linear relationships between absorptive capacity and the extent of spillovers.

In order to test Hypothesis 2, our main explanatory variable is the *quality of institutions* in the host economy and its quadratic term. We rely on several indicators, (1) Rule of Law (RL), from the Worldwide Governance Indicators^9^ (WGI), captures perceptions of the extent to which agents have confidence in and abide by the rules of society, and in particular the quality of contract enforcement, property rights, the police, and the courts, as well as the likelihood of crime and violence. (2) Control of Corruptions (CC), from WGI, captures perceptions of the extent to which public power is exercised for private gain, including both petty and grand forms of corruption, as well as “capture” of the state by elites and private interests. (3) Property Rights (PR), from the World Economic Forum^10^, as part of the global competitiveness indicators based on surveys. In this case, the question is: “In your country, to what extent are property rights, including financial assets, protected? [1 = not at all; 7 = to a great extent]”. Due to the high correlation between indicators, we treat them as alternative measures to check the robustness of our results.

Finally, we control for several other host characteristics at the country, city and FDI project levels. At the level of the country, we control for the *size and development* of the host country, using the GDP and GDP per capita; i*nflation*, as the GDP deflator considering the prices of all goods and services produced; and *international openness* as the flows of inward FDI collected from the World Bank. We also control for the *labor market* using the unemployment rate modelled by the International Labor Organization, and the *geographical distance* between home and host countries (capital to capital) gathered from CEPII.

At the level of the city, we computed two measures to capture the level of *agglomeration economies*: the number of firms in the city in the same sector and the number of firms in other sectors, from Orbis. Finally, we include a control at the level of the FDI project to account for the *type of industry* (NACE 26 is the only one classified as high tech among the four, and the others are medium-high tech) and the *type of the FDI project* (Greenfield, as a dummy equals f the investment project is a greenfield, instead of an M&A deal, from CBI). Year fixed effects are also included. All explanatory variables for the second stage are determined based on the year of the investment, which is the year preceding the spillover parameters (the dependent variable in the second stage).

Table 1 reports the list of (first- and second-stage) variables and descriptive statistics. Table A.5 in the online appendix reports the correlation matrix.

**Table 1:** List of variables and descriptive statistics, first and second stage

Variable	Level	Source	Obs	Mean	Std. Dev
*First stage*					
DV: labor productivity	recipient	Orbis	309,131	11.83	1.26
*FDI projects*					
FDI project value	Project/city	CBI	309,131	16.66	1.57
Capital value other FDI projects	Project/city	CBI	309,131	18.18	5.17
Share of other FDI projects	Project/city	CBI	309,131	0.78	0.32
*City characteristics*					
No. firms in sector-city	City	Orbis	309,131	6.64	0.95
No. firms in other sectors-city	City	Orbis	309,131	8.05	0.75
Tot. assets in city-sector	City	Orbis	309,131	23.70	3.49
*Firm characteristics*					
Firm age	recipient (local firms)	Orbis	309,131	2.34	0.78
Firm size: large	recipient (local firms)	Orbis	309,131	0.41	0.49
Firm size: small	recipient (local firms)	Orbis	309,131	0.23	0.42
*Second stage*					
*Institutions*					
Rule of law	Country	WGI, World Bank	1,266	0.09	0.74
Control of corruption	Country	WGI, World Bank	1,266	0.07	0.85
Property rights	Country	World Economic Forum	1,259	4.71	0.74
*City characteristics*					
Aggreg. Labprod in the host city	City	Orbis	1,266	12.50	1.23
No. firms in sector-city	City	Orbis	1,266	2.39	1.87
No. firms in other sectors-city	City	Orbis	1,266	3.70	1.83
*Country characteristics*					
GDP	Country	World Bank	1,266	28.21	1.50
GDP per capita	Country	World Bank	1,266	9.13	1.08
Unemployment rate	Country	World Bank	1,266	5.03	3.42
Inflation, GDP deflator	Country	World Bank	1,266	2.46	3.58
IFDI	Country	World Bank	1,266	24.66	1.40
Geo distance (capital to capital)	Country	CEPII	1,266	8.94	0.51
*Project level*					
Type: Greenfield	Project/city	CBI	1,266	0.92	0.27

## Results

### Results – first stage

The first stage of our analysis estimates project-level spillover effects on labor productivity of domestic firms in host cities in emerging market economies receiving FDI from developed economies. These project-specific spillover estimates (i.e., coefficients that vary by FDI project) are then used as dependent variables in the second stage, linking host-country and city heterogeneity and institutions with the extent of spillovers.

Table 2 displays the results of the first stage of spillover analysis, based on a mixed-effect model with random parameters (Eq. 3), with the log of labor productivity at the local firm level as the dependent variable. We observe a positive, and statistically significant, mean value of the key explanatory variable in this model - the USD value of capital expenditure in the focal FDI project (*FDI project value* in Table 2). However, significant heterogeneity exists among FDI projects as shown by the statistically significant standard deviation. Figure A.1 in the Online Appendix graphically shows this by plotting the kernel density distribution of the estimated FDI project level parameters of FDI spillovers. Almost all observations of spillover coefficients take an estimated value that is larger than zero, but there is significant variation in the estimated spillovers by focal FDI project. This is consistent with the expectation that spillovers are context-specific: depend on local absorptive capacity (Girma, 2005; Castellani et al., 2024), motivation of FDI, ownership share of FDI in local affiliates and local institutional development (Meyer & Sinani, 2009; Bhaumik et al., 2019). We investigate the factors that explain this variation.

**Table 2:** First stage: estimating productivity spillovers of focal FDI project in the host City, multilevel mixed-effect model

DV: labor productivity	Mod_1	
	Mean	Std. Dev
FDI project value	0.0163***	0.0188***
*se*	(0.0057)	(0.0187)
*pvalue*	([0.0045])	([0.0001])
Capital value other FDI projects	0.0003	
	(0.0023)	
	([0.8803])	
Share of other FDI projects	0.2046***	
	(0.0440)	
	([0.0000])	
Firm age	0.2789***	
	(0.0031)	
	([0.0000])	
Firm size: large	0.1386***	
	(0.0050)	
	([0.0000])	
Firm size: small	0.2332***	
	(0.0070)	
	([0.0000])	
No. firms in sector-city	− 0.0125***	
	(0.0045)	
	([0.0050])	
No. firms in other sectors-city	0.0444***	
	(0.0084)	
	([0.0000])	
Tot. assets in city-sector	0.0191***	
	(0.0016)	
	([0.0000])	
Contant	6.0585***	0.4349***
	(1.1878)	(0.2324)
	([0.0000])	([0.1192])
Random-effects parameters (Project level)		
Corr (Project value, cons)	− 0.9389***	
	(0.0381)	
	([0.0000])	
Std dev (Residual, Total)	1.1674***	
	(0.0015)	
	([0.0000])	
Year fixed effects	Yes	
Country fixed effects	Yes	
Industry fixed effects	Yes	
No. of obs	309,131	
Log-pseudolikelihood	− 487204.02	
p_value comparison test (Multilevel vs. OLS)	0.0000	
Covariance	unstructured	
Sectors (recepients)	24-25-26-27-28-29-33	
MNE sectors (Inward FDI)	26-27-28-29	

The dependent variable is labor productivity. Standard errors in parenthesis below point estimates, p-values in square brackets ([]) below the standard errors. Asterisks denote confidence levels: *p < 0.10, **p < 0.05 and ***p < 0.01. The random parameter model is estimated using the ‘mixed’ package (StataCorp 2013) in Stata 14 and 16, with the covariance(unstructured) option which allows for all variances and covariances to be distinct, and the correlation between random slopes and intercept

Controlling for other FDI projects in the city is important in our first stage model, to not attribute the effects of other projects in the city to the focal one. The share of other FDI projects in the city is a significant factor for local firms’ productivity, in addition to the focal FDI project itself. We further control for firm size, age, and agglomeration-diversification effects.

We observe from Table 2 that small and large firms have higher productivity than medium-sized firms, and older firms have higher productivity. We further show that the agglomeration and diversification effects at the sector-city level matter. Firms in larger sectors, in terms of total assets, have higher productivity. Firms operating in cities with more firms in other sectors have also higher productivity. This may reflect the diversification-related benefits such as knowledge transfer in the form of Jacobian spillovers (Beaudry & Schiffauerova, 2009).

### Results – second stage

This section investigates empirically the heterogeneity of FDI spillovers and tests Hypotheses 1–2, focusing on the roles of the interaction of institutional quality, absorptive capacity, and MNE strategy in shaping the knowledge transfer from MNEs to the host economy. The novel addition to the prior literature links the role of MNE strategy to integrate or not with networks of firms in the host economy. This enables us to explain why spillovers may vary and why empirical studies often find mixed evidence on spillovers, with the results depending on the context of FDI and host economy (e.g., literature reviews in Bhaumik et al., 2019; Keller, 2021).

Our following regression models use the levels and squared terms of absorptive capacity and institutional variables of the host economy as key explanatory variables of the FDI project-specific spillover parameters estimated from the first stage (in Sect. 5.1). We investigate whether there is an optimal level of institutional quality and absorptive capacity that maximizes the level of spillovers, as suggested in Hypotheses 1–2.

**Table 3:** Second stage: exploring the heterogeneity of FDI productivity spillovers across FDI projects and host locations (cities)

	Mod 1	Mod 1a	Mod 2	Mod 2a	Mod 3	Mod 3a	Mod 4	Mod 4a
	Abs. Capacity (ABC)	ABC squared	Rule of Law (RL)	RL squared	Control of Corruption (CC)	CC squared	Property Rights (PR)	PR squared
Aggreg. Labprod in the host city	− 0.0014***	0.0025***	0.0026***	0.0027***	0.0026***	0.0028***	0.0027***	0.0028***
	(0.0002)	(0.0006)	(0.0006)	(0.0006)	(0.0006)	(0.0006)	(0.0006)	(0.0006)
	([0.0000])	([0.0000])	([0.0000])	([0.0000])	([0.0000])	([0.0000])	([0.0000])	([0.0000])
Aggreg. Labprod in the host city sq		− 0.0002***	− 0.0002***	− 0.0002***	− 0.0002***	− 0.0002***	− 0.0002***	− 0.0002***
		(0.0000)	(0.0000)	(0.0000)	(0.0000)	(0.0000)	(0.0000)	(0.0000)
		([0.0000])	([0.0000])	([0.0000])	([0.0000])	([0.0000])	([0.0000])	([0.0000])
Rule of law			− 0.0004	0.0013				
			(0.0005)	(0.0008)				
			([0.4971])	([0.1273])				
Rule of law squared				− 0.0020***				
				(0.0008)				
				([0.0095])				
Control of corruption					− 0.0001	0.0016**		
					(0.0006)	(0.0007)		
					([0.8844])	([0.0262])		
Control of corruption squared						− 0.0019***		
						(0.0005)		
						([0.0003])		
Property rights							− 0.0007	0.0075
							(0.0006)	(0.0054)
							([0.2674])	([0.1657])
Property rights squared								− 0.0009
								(0.0006)
								([0.1283])
Controls								
*Host city level*								
No. firms in sector-city	− 0.0006**	− 0.0007**	− 0.0007**	− 0.0005*	− 0.0007**	− 0.0006**	− 0.0006**	− 0.0006**
	(0.0003)	(0.0003)	(0.0003)	(0.0003)	(0.0003)	(0.0003)	(0.0003)	(0.0003)
	([0.0311])	([0.0117])	([0.0149])	([0.0724])	([0.0123])	([0.0225])	([0.0207])	([0.0301])
No. firms in other sectors-city	0.0010***	0.0012***	0.0012***	0.0011***	0.0012***	0.0011***	0.0012***	0.0011***
	(0.0003)	(0.0003)	(0.0003)	(0.0003)	(0.0003)	(0.0003)	(0.0003)	(0.0003)
	([0.0002])	([0.0000])	([0.0000])	([0.0000])	([0.0000])	([0.0001])	([0.0000])	([0.0000])
*Host Country*								
GDP	− 0.0004	− 0.0002	− 0.0003	− 0.0011**	− 0.0002	− 0.0012**	− 0.0004	− 0.0009*
	(0.0003)	(0.0003)	(0.0004)	(0.0005)	(0.0004)	(0.0005)	(0.0004)	(0.0005)
	([0.1963])	([0.5320])	([0.3746])	([0.0184])	([0.5566])	([0.0101])	([0.2718])	([0.0639])
GDP per capita	0.0003	0.0005*	0.0006*	0.0008**	0.0005	0.0006*	0.0007**	0.0007*
	(0.0002)	(0.0002)	(0.0003)	(0.0003)	(0.0003)	(0.0003)	(0.0004)	(0.0004)
	([0.2965])	([0.0616])	([0.0551])	([0.0108])	([0.1433])	([0.0791])	([0.0476])	([0.0562])
Unemployment rate	0.000	0.000	0.000	0.000	0.000	0.000	0.000	0.000
	(0.0001)	(0.0001)	(0.0001)	(0.0001)	(0.0001)	(0.0001)	(0.0001)	(0.0001)
	([0.5534])	([0.8432])	([0.8508])	([0.7212])	([0.8504])	([0.5536])	([0.8121])	([0.8193])
Inflation, GDP deflator	0.0003***	0.0003***	0.0003***	0.0004***	0.0003***	0.0004***	0.0003***	0.0003***
	(0.0001)	(0.0001)	(0.0001)	(0.0001)	(0.0001)	(0.0001)	(0.0001)	(0.0001)
	([0.0000])	([0.0000])	([0.0000])	([0.0000])	([0.0000])	([0.0000])	([0.0001])	([0.0001])
IFDI	0.0001	0.000	0.000	0.0009*	0.000	0.0009**	0.0001	0.0005
	(0.0003)	(0.0003)	(0.0003)	(0.0005)	(0.0003)	(0.0004)	(0.0003)	(0.0004)
	([0.7163])	([0.8672])	([0.9789])	([0.0527])	([0.9291])	([0.0242])	([0.7085])	([0.2286])
Geo distance (capital to capital)	− 0.0020***	− 0.0021***	− 0.0020***	− 0.0020***	− 0.0021***	− 0.0020***	− 0.0020***	− 0.0020***
	(0.0004)	(0.0004)	(0.0004)	(0.0004)	(0.0004)	(0.0004)	(0.0004)	(0.0004)
	([0.0000])	([0.0000])	([0.0000])	([0.0000])	([0.0000])	([0.0000])	([0.0000])	([0.0000])
*Project level*								
Type: Greenfield	− 0.0004	− 0.0005	− 0.0005	− 0.0007	− 0.0005	− 0.0006	− 0.0005	− 0.0006
	(0.0008)	(0.0008)	(0.0008)	(0.0008)	(0.0008)	(0.0008)	(0.0008)	(0.0008)
	([0.6114])	([0.5657])	([0.5144])	([0.3867])	([0.5575])	([0.4569])	([0.5186])	([0.4496])
Industry NACE 26, HT	0.0017**	0.0018***	0.0018***	0.0017**	0.0018***	0.0017**	0.0018**	0.0018**
	(0.0007)	(0.0007)	(0.0007)	(0.0007)	(0.0007)	(0.0007)	(0.0007)	(0.0007)
	([0.0199])	([0.0092])	([0.0097])	([0.0166])	([0.0093])	([0.0161])	([0.0102])	([0.0104])
Industry NACE 28, MHT	0.0005	0.0006	0.0006	0.0005	0.0006	0.0005	0.0006	0.0005
	(0.0008)	(0.0007)	(0.0007)	(0.0007)	(0.0007)	(0.0007)	(0.0007)	(0.0007)
	([0.5465])	([0.3907])	([0.4216])	([0.5313])	([0.3971])	([0.4775])	([0.4149])	([0.4653])
Industry NACE 29, MHT	− 0.0001	− 0.0001	− 0.0001	− 0.0001	− 0.0001	− 0.0001	− 0.0001	− 0.0002
	(0.0008)	(0.0007)	(0.0008)	(0.0008)	(0.0008)	(0.0008)	(0.0008)	(0.0008)
	([0.8548])	([0.9309])	([0.8934])	([0.8861])	([0.9200])	([0.8806])	([0.8560])	([0.7867])
Constant	0.0541***	0.0318***	0.0322***	0.0327***	0.0318***	0.0353***	0.0329***	0.0197
	(0.0078)	(0.0083)	(0.0084)	(0.0083)	(0.0083)	(0.0084)	(0.0089)	(0.0124)
	([0.0000])	([0.0001])	([0.0001])	([0.0001])	([0.0001])	([0.0000])	([0.0002])	([0.1126])
Year fixed effects	Yes	Yes	Yes	Yes	Yes	Yes	Yes	Yes
No of obs	1266	1266	1266	1266	1266	1266	1259	1259
R-squared	0.076	0.108	0.109	0.113	0.108	0.118	0.109	0.111
R-squared adjusted	0.064	0.095	0.095	0.099	0.095	0.103	0.096	0.097

Note. The dependent variable is the FDI project-specific spillover effect from the first stage (Table 2). We predict project-specific coefficients using the post-estimation command ‘predict’, including reffects option within the mixed post-estimation and related ‘mixed’ packages in Stata 14 and 16 (StataCorp 2013). Standard errors (in parenthesis below the parameter estimates) are computed based on Hornstein and Greene (2012). P-values in square brackets below the standard errors. Asterisks denote confidence levels: *p < 0.10, **p < 0.05 and ***p < 0.01

**Fig. 2:**
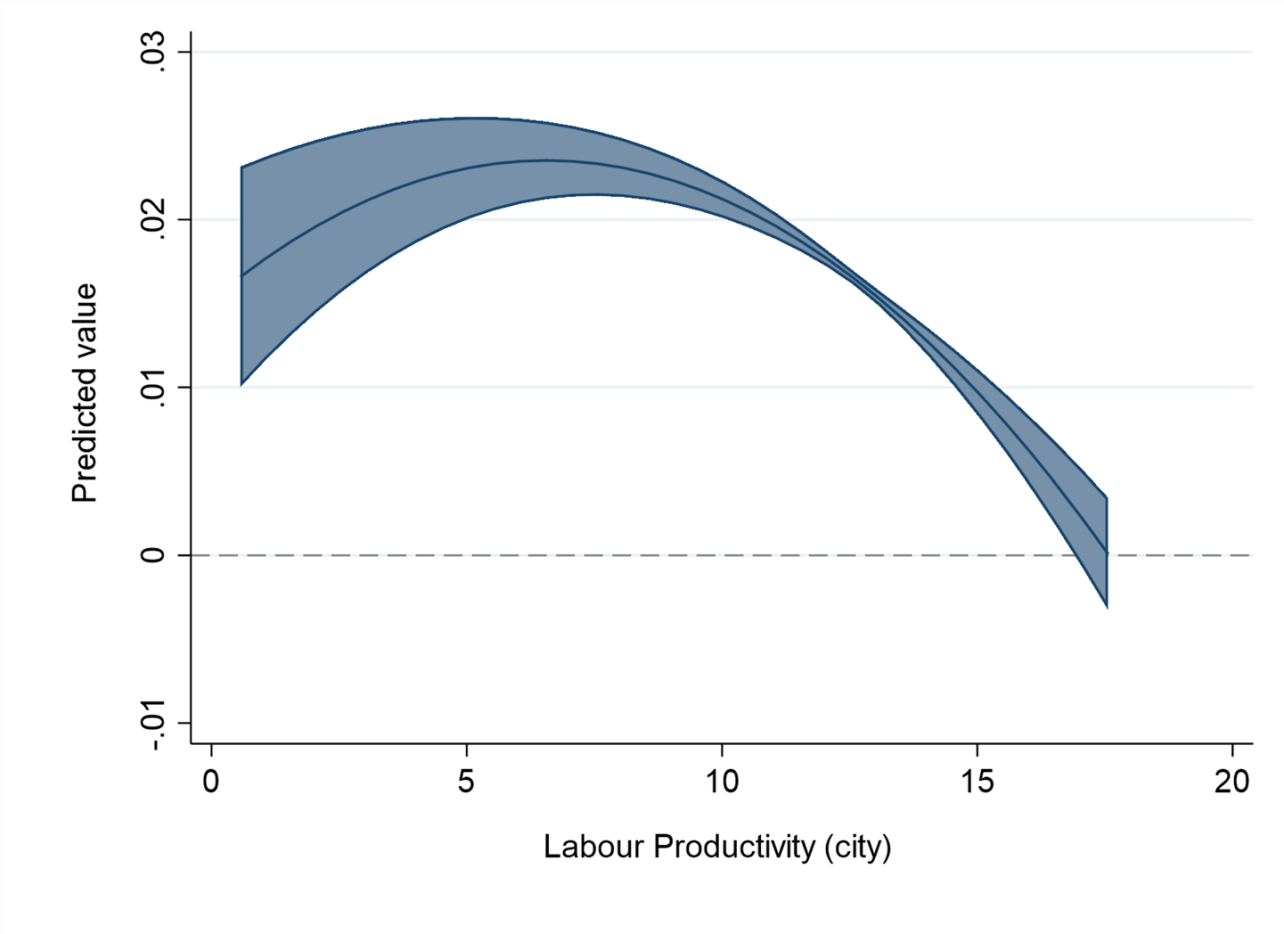
The effect of aggregate labor productivity of the host city on the FDI spillover returns. The figure is created from mod. 1a in Table 3, using the values of aggregate labor productivity and keeping the other variables at their mean values (margins at the means of covariates). The line represents the average marginal effect of absorptive capacity on the linear probability of FDI spillover returns (Y axis) for different levels of labor productivity (X axis). The upper-bound and the lower-bound lines represent the confidence interval (at 95% level) for the represented marginal effects. The dashed line represents 0, i.e. the zone where marginal effects are not statistically significant at 10% p-value.

We proxy the absorptive capacity with the aggregate labor productivity of local firms in the city. We observe an inverted U-shaped relationship between the absorptive capacity and the extent of spillovers (see Mod. 1a in Table 3; Fig. 2). Thus, there is an optimal level of absorptive capacity that maximizes the benefits of FDI, after that spillovers returns start decreasing. We note that the optimal level (i.e., a turning point) in the inverted U-shaped relationship in Fig. 2 is reached at a lower level of productivity than the mean productivity level of the sample. Thus, the majority of observations are located at the downward-sloping part of the curve in Fig. 2: in the case of these host cities and FDI projects the high local absorptive capacity (i.e., high competitiveness of local firms) is limiting positive spillovers. This supports our Hypothesis 1 and emphasizes the need to explore the non-linear effects of institutions and local absorptive capacity on FDI spillover generation, to advance a traditional view that a high level of institutions fosters spillovers and a low level limits them.

Turning to the quality of institutions (Hypothesis 2), we estimate regression models assuming that the effect of institutions is linear and monotonic, with the set of controls as outlined in Sect. 4.2.2. None of the institutional variables turns out to be significant in these simple spillover models (see Mod. 2, 3 and 4 in Table 3).

**Fig. 3:**
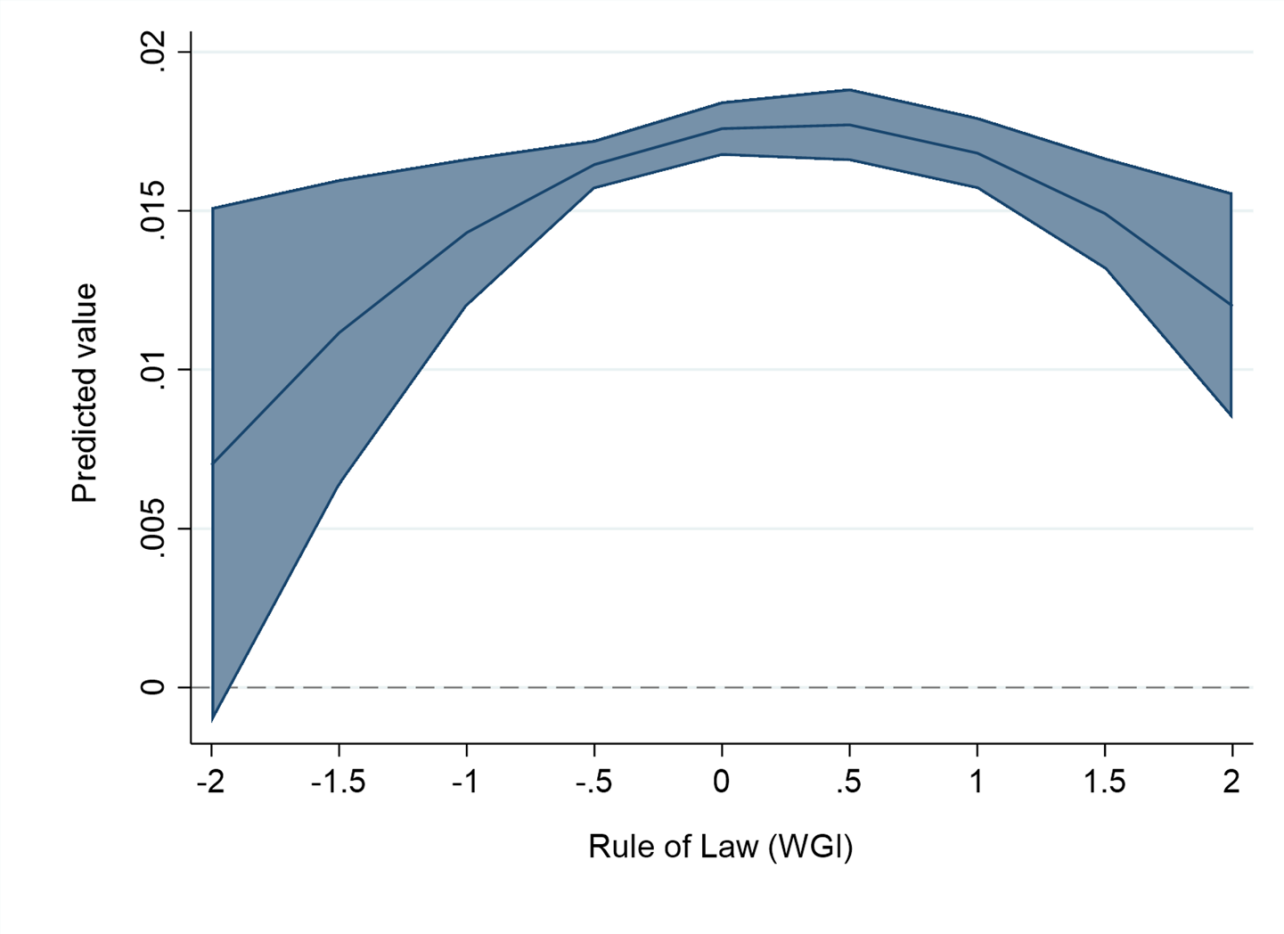
The effect of Rule of law on the FDI spillover returns. The figure is created from mod. 2a in Table 3, using the values of Rule of law and keeping the other variables at their mean values (margins at the means of covariates). The line represents the average marginal effect of institutions on the linear probability of FDI spillover returns (Y axis) for different levels of the rule of law (X axis). The upper-bound and the lower-bound lines represent the confidence interval (at 95% level) for the represented marginal effects. The dashed line represents 0, i.e. the zone where marginal effects are not statistically significant at 10% p-value.

**Fig. 4:**
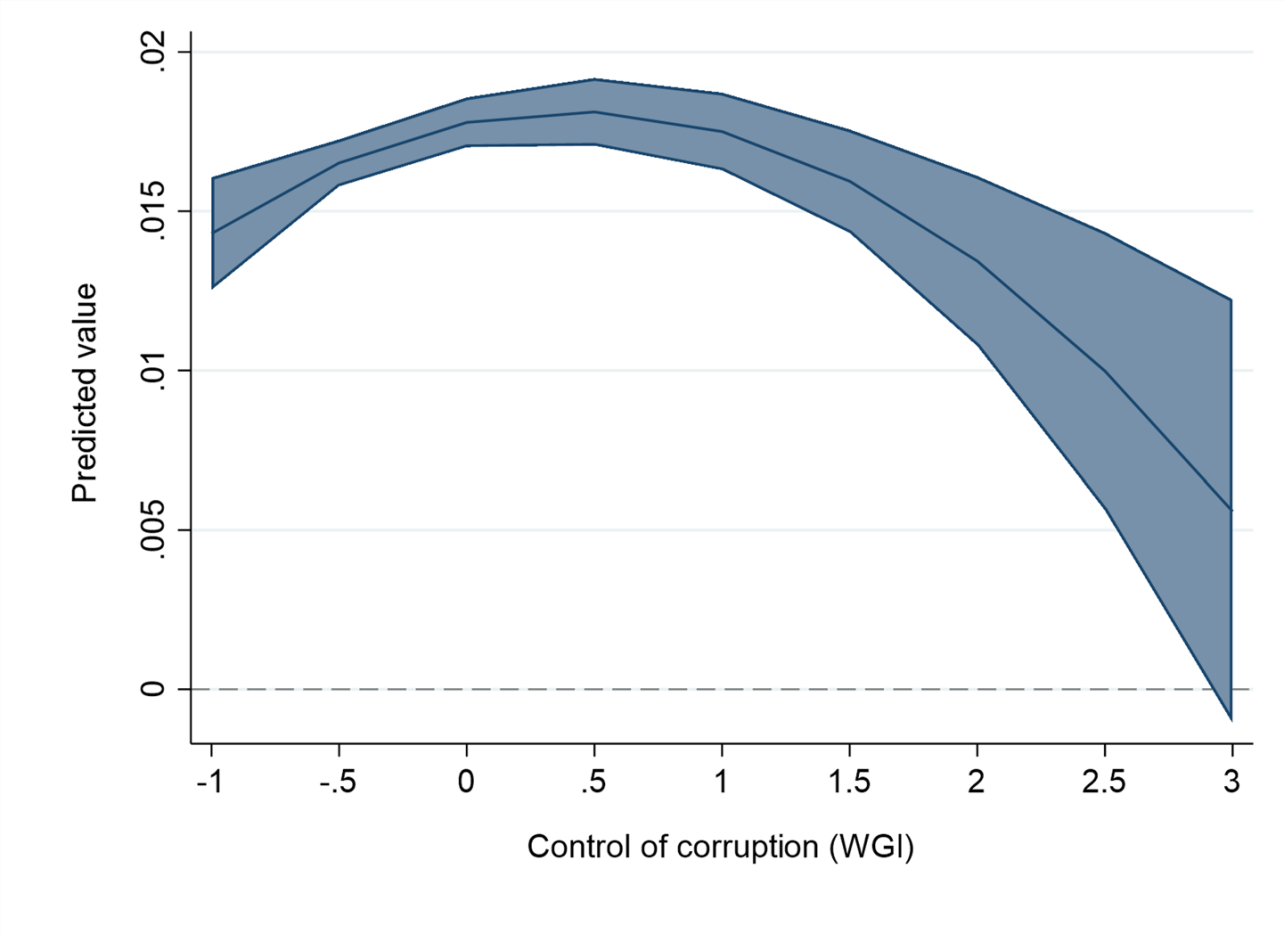
The effect of Control of corruption on the FDI spillover returns. The figure is created from mod. 3a in Table 3, using the values of Control of corruption and keeping the other variables at their mean values (margins at the means of covariates). The line represents the average marginal effect of institutions on the linear probability of FDI spillover returns (Y axis) for different levels of control of corruption (X axis). The upper-bound and the lower-bound lines represent the confidence interval (at 95% level) for the represented marginal effects. The dashed line represents 0, i.e. the zone where marginal effects are not statistically significant at 10% p-value.

**Fig. 5:**
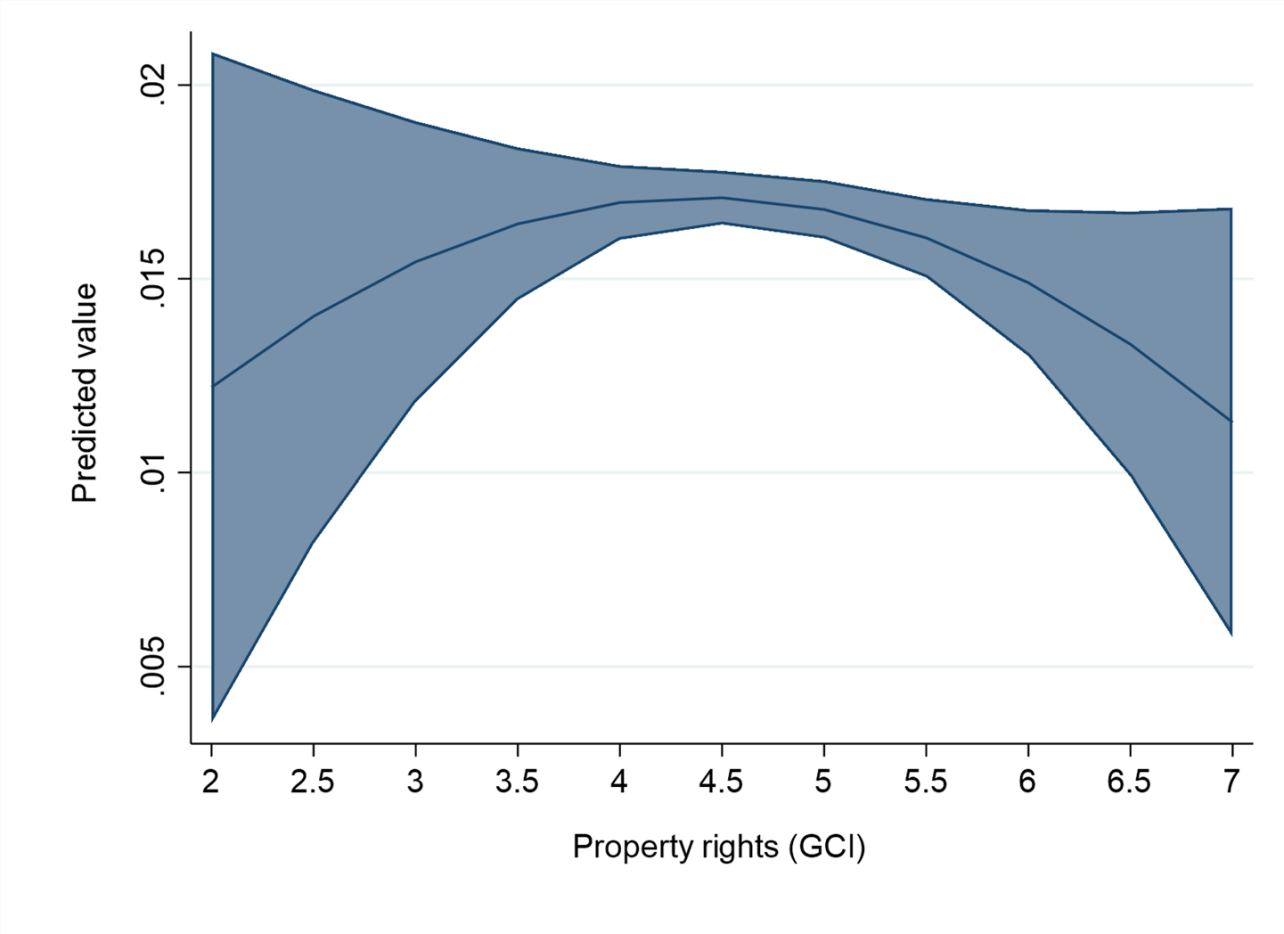
The effect of Property rights on the FDI spillover returns. The figure is created from mod. 4a in Table 3, using the values of Property rights and keeping the other variables at their mean values (margins at the means of covariates). The line represents the average marginal effect of institutions on the linear probability of FDI spillover returns (Y axis) for different levels of property rights (X axis). The upper-bound and the lower-bound lines represent the confidence interval (at 95% level) for the represented marginal effects.

These findings hide significant non-linearity. Once we add the quadratic term of each institutional variable to the second-stage models, we find evidence of a statistically significant and inverted U-shaped relationship between institutional quality and the magnitude of spillovers (see Table 3; Figs. 3, 4 and 5). This means that a ‘medium’ level of institutional development maximizes the spillovers of FDI, compared to both ‘low’ and ‘high’ levels.

The inverted U-shaped effect is there in the case of all the studied institutional variables: rule of law (Mod. 2a in Table 3; Fig. 3), control of corruption (Column 3a in Table 3; Fig. 4), and the level of protection of property rights – although less precisely estimated (Column 4a in Table 3; Fig. 5). In the case of the rule of law, control of corruption and protection of property rights, the levels of institutional variables that maximize spillovers are close to the mean value of these institutional variables in our estimation sample. For example, in the case of the rule of law indicator, the turning point where the positive effects of FDI projects are maximized is at value 0.3. The mean value of the same variable in the estimation sample is close to that, with a mean value of 0.09. In general, for host countries and cities with below-average institutional development levels, improvement in the rule of law, corruption control and property rights protection are spillover-enhancing. For them, the implications of improved institutions follow the conventional view of the positive role of institutions in knowledge transfer from FDI. However, the opposite result holds for host cities with a higher than average level of institutions, supporting our Hypothesis 2.

All regressions include controls at the city, country, and project level. The number of firms in the city is negatively and statistically significantly associated with spillovers, suggesting that highly specialized cities benefit less from FDI. The indicator of diversification (Jacobian spillovers) - the number of firms in other sectors - is associated with increased spillovers (Beaudry & Schiffauerova, 2009; Castellani et al., 2024). Among the host country variables, GDP per capita, inflation and distance between the capitals of the host and home country are significant. Distance matters: destinations that are farther from the home country of investors gain less through spillovers. This reflects the standard findings from gravity models of FDI and trade (e.g., Baier, 2019). We further show that there is no difference in spillovers between greenfield FDI and M&As. Finally, we show industry-specific effects: the NACE high-technology sector 26 (Manufacture of computer, electronic and optical products) has stronger spillovers compared to the other medium high-technology sectors covered in our analysis (NACE 28 and 29).

## Conclusions

This study aims to shed additional light on the role of institutions in facilitating knowledge spillovers in the context of MNEs. The existing body of literature has mainly investigated the linear relationship between favorable institutional quality and knowledge transfer, along with the sub-national level of absorptive capacity of local firms. This paper presents more complex interrelations between the host environment and MNE strategy, therefore spillovers, proposing an inverted U-shaped relationship: MNEs might hesitate to transfer knowledge to subsidiaries not only in environments with poor institutional quality, as traditionally discussed, but also in those characterized by high institutional quality.

The evidence presented is consistent with the proposed conceptual model, which emphasizes the role of MNE strategy in the interaction with local institutions and absorptive capacity. At low levels of institutional development, MNEs would endeavor to limit spillovers and knowledge transfer to the host economy because of concerns concerning the lack of IP protection, limited enforceability of contracts and weak courts of law, to avoid the appropriation of MNE’s intangibles by local firms. At low starting levels of institutional quality in a host city, an improvement in institutions would guide strategic decisions of the MNE towards lesser use of defensive strategies and more integration of the MNE with local firms, consequently a likely increase in spillovers.

At high levels of quality of local institutions, the local firms have built strong capabilities (Liu, 2008; Meyer & Sinani, 2009), including complementary knowledge assets and strong absorptive capacity in the future (Zahra & George, 2002). These complementary knowledge resources make them more able to engage successfully in innovation and building of intangibles, as suggested also by the profiting-from-innovation framework in Teece (1986), to absorb external knowledge (Cohen & Levinthal, 1989) and combine it with their own knowledge investments, consequently making them potentially significant competitors for MNEs.

In that case, multinational firms are deterred from becoming embedded in the local economy due to concerns of high levels of local, regional (and in some cases potentially also global) competition. The strong capabilities of incumbents for building their own intangibles and learning from external sources leads to a higher risk of spillovers and consequently to a more defensive strategy of the MNE to limit knowledge transfer. These considerations can for example also affect the decision of the firm whether to produce the knowledge-intensive inputs within the boundary of the MNE or to source these from local external partners.

As a result of the two types of opposing effects, there is going to be an optimal level of institutional quality that maximizes the spillovers to the host city. The results of our empirical analysis strongly support this proposition. The empirical results suggest that the best response of the MNE would be to integrate less with the host country market both when the host country’s institutional quality and local absorptive capacity are ‘High’ and ‘Low’, and integrate most when the host country’s institutional quality is ‘Medium’.

This brings important contributions and policy implications. In terms of maximizing the benefits from inward investment, this highlights the need to better understand the relationships between firm-specific assets and local institutions. One may need for example to think in terms of appropriate institutional level, rather than an absolute improvement in institutional quality as articulated in the so-called Washington consensus. As an illustration, strong IPR protection boosts local productivity, and attracts FDI, but may limit the scale of spillovers from that investment. If local policy is focused on investment leading endogenous growth, then a more nuanced policy towards patenting may be required, or countries may seek to link FDI incentives to knowledge sharing, effectively moving the turning point in the relationship between spillovers and institutional quality to the right.

Similarly, our results suggest that small changes in IP protection can influence firm-level investment decisions that may limit a country’s ability to participate in global value chains. Collectively, our results suggest that where institutions are weak, improving these has the greatest return, in terms of FDI attraction, inflows of knowledge and in terms knowledge transfer from inward investors to domestic firms. Our results suggest that above all, improving areas of institutional weakness has the greatest benefit. In devolved economies, this may require intervention to improve knowledge ecosystems at a local level, as well as nationally focused initiatives targeting certain sectors. Similarly, this raises some interesting questions for entrepreneurship scholars, regarding the best mechanisms for fostering productivity growth in the types of firms that may appropriate this knowledge. This traditional literature in this area, see for example Mansury et al., (2008), focuses on innovation as the main source of productivity growth, and while our findings are consistent with this, one may also suggest a wider set of interventions to support local firms. This may include programs to assist firms in becoming suppliers to MNEs, and access to finance to boost productivity.

Our results suggest that an interesting avenue for research could be the interplay between institutions and local productivity for maximizing, not FDI flows, or even knowledge transfer between inward investors and local firms, but the overall benefits of FDI. We have demonstrated that a key consideration for a multinational firm in terms of its decision to transfer knowledge to an emerging economy is the nature of institutions that it will encounter and that this relationship is non-linear. At the same time, it is reasonable to assume that the level of local absorptive capacity is in part a function of past institutions. For a country then seeking to maximize the gains from attracting inward FDI, this represents a nontrivial trade-off between the “optimum” level of institutional quality that attracts the highest levels of foreign knowledge, and the potential trade-off between this and a potentially greater level of institutional quality that facilitates higher productivity growth in the local economy. This calls for future research on the complex interrelationships between institutions and absorptive capacity, MNE strategies and FDI spillovers.

Furthermore, we adopt a novel methodological approach that enables a more granular investigation of spillovers, beyond the average effects of FDI presence in a sector (see also Castellani et al., 2024), or in downstream or upstream sectors, as conventionally investigated in standard analysis of FDI spillovers (e.g., in seminal empirical papers such as Aitken & Harrison, 1999; Javorcik, 2004). A recent study on FDI spillovers using transaction-level data in Costa Rica by Alfaro-Urena et al. (2022) highlights significant limitations of standard FDI spillover analyses, which fail to capture the heterogeneous effects of FDI. Similarly, Keller (2021) underscores the shortcomings of conventional approaches to studying spillovers. Our analysis focuses on contextual factors related to the institutional environment, explaining variations in FDI spillovers. However, this empirical approach allows to control for a rich set of factors, at different levels, namely project, country, region and firm levels. Future studies could extend this research by exploring additional factors explaining the variation of FDI spillovers, and deepening the investigation on the characteristics of MNEs. Additionally, we present new evidence on the relationship between the quality of institutions and the magnitude of FDI spillovers. However, this relationship is inherently complex, as institutions are intertwined with several factors that may deter MNE integration. Future research should further investigate these mechanisms. Finally, our analysis relies on cross-sectional data and we cannot observe the dynamic over time on institutional quality and FDI: a longitudinal study could examine how changes in institutional quality over time affect spillovers, and observe the phenomenon across diverse sectors of the economy. Our study focuses on the role of formal institutions, future research can explore the role of informal institutions which are often critical in emerging countries.

## Electronic supplementary material

Below is the link to the electronic supplementary material.

Supplementary Material 1

## Data Availability

Part of the research data used in this study is obtained from Orbis Crossborder Investment and Orbis — Moody’s. Restrictions apply to the availability of these data, which were used under license. Data access is subject to the permission of the data provider.

## Footnotes

1 This is not to suggest that informal institutions are not useful. However, as Stiglitz (2000) argues, “[a]s a society develops economically, its social capital [that helps it to cope with moral hazard and incentive problems] must adapt as well, allowing the interpersonal networks to be replaced with the formal institutions of a market-based economy” (pp. 59).

2 While Coasian institutions include both property rights and those ensuring sanctity and enforceability of contracts, it has been argued that property rights have a “first order” effect on economic outcomes such as growth, while “contracting institutions” only matter for financial intermediation (Acemoglu & Johnson, 2005). This is consistent with the discussion about institutions in the IB literature.

3 Data from the World Bank suggests that the property rights index has much lower scores for the BRICS countries (Brazil 55.80; Russia 48.70; India 55.40; China 46.70 and South Africa 67.60) than for developed countries such as the USA (79.30); UK (92.20); France (84.00), Germany (81.00) and Japan (86.00).

4 We assume that $\:{\beta\:}_{i}$ is normally distributed, namely $\:{\beta\:}_{i}$~ *N* ($\:\beta\:$,$\:\:\sigma\:$).

5 For more details, please see https://unctadstat.unctad.org/EN/Classifications/DimCountries_All_Hierarchy.pdf.

6 For more details on the classification, please see https://ec.europa.eu/eurostat/statistics-explained/index.php?%20title=Glossary:High-tech_classification_of_manufacturing_industries.

7 More specifically, 2-digit NACE Rev. 2 code 26-Manufacture of computer, electronic and optical products, 27-Manufacture of electrical equipment, 28-Manufacture of machinery and equipment n.e.c., 29-Manufacture of motor vehicles, trailers and semi-trailers. The companies can report this sector as primary or secondary sector.

8 Moreover, in order to include the project in the sample, we need to be able to collect the relevant information of local firms located in the destination cities of our focal investments (second step), this is crucial to estimate our first stage.

9 For additional details: https://info.worldbank.org/governance/wgi/.

10 For additional details: https://tcdata360.worldbank.org/indicators/h1cdfe8bd?%20country=BRA&indicator=634&viz=line_chart&years=2007,2017.
