# Supplementary material for: Multinational strategy, institutions and spillovers: the role of institutions in knowledge spillovers in emerging markets
Source: J Technol Transf. 2025 May 31;50(5):2476–508. doi: 10.1007/s10961-025-10230-w (PMC12460489; doi:10.1007/s10961-025-10230-w)
Supplement: Supplementary file 1 — Supplementary Material 1 [file 10961_2025_10230_MOESM1_ESM.docx]

ONLINE APPENDIX

**Multinational strategy, institutions and spillovers:**

**the role of institutions in knowledge spillovers in emerging markets**

Table A.1. Distribution of investment projects, by country of origin (2013-2017)

| **Country of Origin** | **No.** | **%** |
| --- | --- | --- |
| United States of America | 430 | 33.97 |
| Japan | 237 | 18.72 |
| Germany | 200 | 15.8 |
| Switzerland | 73 | 5.77 |
| France | 57 | 4.5 |
| United Kingdom | 45 | 3.55 |
| Netherlands | 42 | 3.32 |
| Finland | 28 | 2.21 |
| Canada | 25 | 1.97 |
| Italy | 25 | 1.97 |
| Sweden | 22 | 1.74 |
| Austria | 16 | 1.26 |
| Denmark | 12 | 0.95 |
| Spain | 12 | 0.95 |
| Ireland | 7 | 0.55 |
| Luxembourg | 7 | 0.55 |
| Australia | 5 | 0.39 |
| Belgium | 4 | 0.32 |
| Bermuda | 4 | 0.32 |
| Norway | 4 | 0.32 |
| Israel | 3 | 0.24 |
| Czech Republic | 2 | 0.16 |
| Belarus | 1 | 0.08 |
| Cyprus | 1 | 0.08 |
| New Zealand | 1 | 0.08 |
| Poland | 1 | 0.08 |
| Portugal | 1 | 0.08 |
| Russian Federation | 1 | 0.08 |
| Total | 1266 | 100 |

Source: Orbis – Cross Border Investment.

Note: we select projects undertaken by MNEs from developed countries, operating in four industries, namely 2-digit NACE code 26-Manufacture of computer, electronic and optical products, 27-Manufacture of electrical equipment, 28-Manufacture of machinery and equipment n.e.c., 29-Manufacture of motor vehicles, trailers and semi-trailers.

Table A.2. Distribution of investment projects, by destination country (2013-2017)

| **Country of Destination** | **No.** | **%** |
| --- | --- | --- |
| China | 425 | 33.57 |
| India | 180 | 14.22 |
| Mexico | 131 | 10.35 |
| Singapore | 115 | 9.08 |
| UAE | 87 | 6.87 |
| South Korea | 45 | 3.55 |
| Brazil | 44 | 3.48 |
| Malaysia | 38 | 3 |
| Hong Kong | 28 | 2.21 |
| South Africa | 26 | 2.05 |
| Indonesia | 23 | 1.82 |
| Vietnam | 23 | 1.82 |
| Turkey | 21 | 1.66 |
| Saudi Arabia | 18 | 1.42 |
| Colombia | 15 | 1.18 |
| Chile | 11 | 0.87 |
| Niger | 7 | 0.55 |
| Qatar | 5 | 0.39 |
| Egypt | 4 | 0.32 |
| Pakistan | 3 | 0.24 |
| Peru | 3 | 0.24 |
| Philippines | 3 | 0.24 |
| Bangladesh | 2 | 0.16 |
| Jordan | 2 | 0.16 |
| Sri Lanka | 2 | 0.16 |
| Argentina | 1 | 0.08 |
| Bahrain | 1 | 0.08 |
| Ecuador | 1 | 0.08 |
| Kuwait | 1 | 0.08 |
| Paraguay | 1 | 0.08 |
| Total | 1266 | 100 |

Source: Orbis – Cross Border Investment.

Note: we select projects undertaken by MNEs from developed countries, operating in four industries, namely 2-digit NACE code 26-Manufacture of computer, electronic and optical products, 27-Manufacture of electrical equipment, 28-Manufacture of machinery and equipment n.e.c., 29-Manufacture of motor vehicles, trailers and semi-trailers.

Table A.3. List of destination cities, and distribution of projects by city

| City | No. | % | City | No. | % | City | No. | % | City | No. | % | City | No. |
| --- | --- | --- | --- | --- | --- | --- | --- | --- | --- | --- | --- | --- | --- |
| Abu Dhabi | 15 | 1.18 | Chongqing | 20 | 1.58 | Hung Hom | 1 | 0.08 | Midrand | 1 | 0.08 | Shiyan | 2 |
| Agua Prieta | 1 | 0.08 | Chungju | 2 | 0.16 | Hyderabad | 13 | 1.03 | Monclova | 2 | 0.16 | Singapore | 115 |
| Aguascalientes | 7 | 0.55 | Ciudad Juarez | 1 | 0.08 | Icheon | 2 | 0.16 | Monterrey | 12 | 0.95 | Sorocaba | 3 |
| Ahmedabad | 3 | 0.24 | Colombo | 2 | 0.16 | Incheon | 1 | 0.08 | Mumbai | 13 | 1.03 | Surabaya | 1 |
| Al Khobar | 1 | 0.08 | Copiapo | 1 | 0.08 | Indore | 1 | 0.08 | Nagpur | 1 | 0.08 | Suwon | 5 |
| Alberton | 1 | 0.08 | Corregidora | 1 | 0.08 | Irapuato | 3 | 0.24 | Nanchang | 1 | 0.08 | Suzhou | 13 |
| Amman | 2 | 0.16 | Dalian | 11 | 0.87 | Istanbul | 19 | 1.5 | Nanjing | 10 | 0.79 | Taicang | 4 |
| Anseong | 1 | 0.08 | Dammam | 2 | 0.16 | Iztapalapa, Mexico City | 1 | 0.08 | Nantong | 3 | 0.24 | Tangerang | 5 |
| Apodaca | 3 | 0.24 | Delhi | 3 | 0.24 | Jakarta | 17 | 1.34 | New Delhi | 13 | 1.03 | Tangshan | 1 |
| Arteaga | 1 | 0.08 | Dhaka | 2 | 0.16 | Jeddah | 4 | 0.32 | Ningbo | 5 | 0.39 | Thane | 3 |
| Asuncion | 1 | 0.08 | Doha | 5 | 0.39 | Jian | 1 | 0.08 | Ningde | 2 | 0.16 | Tianjin | 11 |
| Aurangabad | 1 | 0.08 | Dongguan | 3 | 0.24 | Jiangmen | 1 | 0.08 | Noida | 8 | 0.63 | Tijuana | 6 |
| Azcapotzalco | 1 | 0.08 | Dubai | 71 | 5.61 | Jiaxing | 2 | 0.16 | Palmira | 1 | 0.08 | Tlajomulco De Zuniga | 2 |
| Bac Ninh | 1 | 0.08 | Durban | 1 | 0.08 | Jinan | 6 | 0.47 | Pasig | 2 | 0.16 | Tlaxcala | 1 |
| Bangalore | 49 | 3.87 | El Marques | 2 | 0.16 | Johannesburg | 14 | 1.11 | Penang | 7 | 0.55 | Toluca | 2 |
| Baoding | 2 | 0.16 | Escobedo | 5 | 0.39 | Johor Bahru | 2 | 0.16 | Petaling Jaya | 7 | 0.55 | Torreon | 1 |
| Barranquilla | 1 | 0.08 | Faridabad | 1 | 0.08 | Juarez | 1 | 0.08 | Pretoria | 1 | 0.08 | Uitenhage | 1 |
| Beijing | 29 | 2.29 | Foshan | 3 | 0.24 | Jundiai | 1 | 0.08 | Puebla | 4 | 0.32 | Vadodara | 3 |
| Betim | 1 | 0.08 | Funza | 1 | 0.08 | Kancheepuram | 1 | 0.08 | Pune | 29 | 2.29 | Visakhapatnam | 1 |
| Bhiwadi | 1 | 0.08 | Fuzhou | 4 | 0.32 | Karachi | 1 | 0.08 | Pyeongtaek | 1 | 0.08 | Wan Chai | 1 |
| Bogota | 10 | 0.79 | Gandhinagar | 1 | 0.08 | Kochi | 1 | 0.08 | Qingdao | 8 | 0.63 | Wuhan | 13 |
| Buenos Aires | 1 | 0.08 | Ghaziabad | 1 | 0.08 | Kolkata | 4 | 0.32 | Queretaro | 15 | 1.18 | Wuxi | 23 |
| Bursa | 2 | 0.16 | Gimpo-si | 1 | 0.08 | Kuala Lumpur | 18 | 1.42 | Quito | 1 | 0.08 | Xi'an | 3 |
| Busan | 5 | 0.39 | Gravatai | 1 | 0.08 | Kuching | 1 | 0.08 | Ramos Arizpe | 1 | 0.08 | Xian | 1 |
| Cairo | 4 | 0.32 | Guadalajara | 8 | 0.63 | Kunming | 1 | 0.08 | Resende | 1 | 0.08 | Xiangyang | 3 |
| Calama | 1 | 0.08 | Guangzhou | 17 | 1.34 | Kunshan | 9 | 0.71 | Rio De Janeiro | 12 | 0.95 | Xiaogan | 1 |
| Campinas | 1 | 0.08 | Guiyang | 1 | 0.08 | Kuwait City | 1 | 0.08 | Riyadh | 11 | 0.87 | Xingtai | 1 |
| Can Tho | 1 | 0.08 | Gurgaon | 6 | 0.47 | Lagos | 7 | 0.55 | SAO Paulo | 2 | 0.16 | Yancheng | 1 |
| Cape Town | 7 | 0.55 | Ha Noi | 6 | 0.47 | Lahore | 2 | 0.16 | Saltillo | 6 | 0.47 | Yantai | 3 |
| Celaya | 3 | 0.24 | Hai Duong | 2 | 0.16 | Langfang | 2 | 0.16 | San Juan Del Rio | 2 | 0.16 | Yichun | 1 |
| Chandigarh | 2 | 0.16 | Hai Phong | 3 | 0.24 | Leon | 3 | 0.24 | San Luis Potosi | 11 | 0.87 | Yongin | 1 |
| Changchun | 1 | 0.08 | Hanam | 1 | 0.08 | Lerma | 2 | 0.16 | Santiago | 9 | 0.71 | Yulin | 1 |
| Changsha | 5 | 0.39 | Hangzhou | 6 | 0.47 | Lima | 3 | 0.24 | Sao Paulo | 19 | 1.5 | Zapopan | 2 |
| Changshu | 3 | 0.24 | Haryana | 1 | 0.08 | Linyi | 1 | 0.08 | Seongnam | 1 | 0.08 | Zhangjiagang | 1 |
| Changwon | 2 | 0.16 | Hebi | 1 | 0.08 | Luoyang | 1 | 0.08 | Seoul | 21 | 1.66 | Zhengzhou | 2 |
| Changzhou | 10 | 0.79 | Hefei | 6 | 0.47 | Makati | 1 | 0.08 | Shah Alam | 3 | 0.24 | Zhenjiang | 2 |
| Chengdu | 10 | 0.79 | Heyuan | 1 | 0.08 | Manama (Al-Manamah) | 1 | 0.08 | Shanghai | 125 | 9.87 | Zhuhai | 5 |
| Chennai | 20 | 1.58 | Ho Chi Minh | 10 | 0.79 | Manaus | 3 | 0.24 | Shaoxing | 1 | 0.08 |  |  |
| Cheongju-SI | 1 | 0.08 | Hohhot | 1 | 0.08 | Medellin | 2 | 0.16 | Sharjah | 1 | 0.08 |  |  |
| Cheung Sha Wan | 1 | 0.08 | Hong Kong | 25 | 1.97 | Mexicali | 1 | 0.08 | Shenyang | 7 | 0.55 |  |  |
| Chihuahua | 3 | 0.24 | Huai’An | 1 | 0.08 | Mexico City | 17 | 1.34 | Shenzhen | 12 | 0.95 | Total | 1,266 |

Table A.4. Distribution of firms by city

| City | No. | % | City | No. | % | City | No. | % | City | No. | % | City | No. | % |
| --- | --- | --- | --- | --- | --- | --- | --- | --- | --- | --- | --- | --- | --- | --- |
| Abu Dhabi | 65 | 0.2 | Chongqing | 437 | 1.32 | Hung Hom | 14 | 0.04 | Midrand | 2 | 0.01 | Shiyan | 30 | 0.09 |
| Agua Prieta | 3 | 0.01 | Chungju | 39 | 0.12 | Hyderabad | 30 | 0.09 | Monclova | 7 | 0.02 | Singapore | 39 | 0.12 |
| Aguascalientes | 18 | 0.05 | Ciudad Juarez | 5 | 0.02 | Icheon | 31 | 0.09 | Monterrey | 260 | 0.78 | Sorocaba | 148 | 0.45 |
| Ahmedabad | 33 | 0.1 | Colombo | 9 | 0.03 | Incheon | 977 | 2.95 | Mumbai | 166 | 0.5 | Surabaya | 6 | 0.02 |
| Al Khobar | 25 | 0.08 | Copiapo | 2 | 0.01 | Indore | 6 | 0.02 | Nagpur | 8 | 0.02 | Suwon | 205 | 0.62 |
| Alberton | 4 | 0.01 | Corregidora | 3 | 0.01 | Irapuato | 9 | 0.03 | Nanchang | 47 | 0.14 | Suzhou | 1,802 | 5.44 |
| Amman | 17 | 0.05 | Dalian | 280 | 0.85 | Istanbul | 7 | 0.02 | Nanjing | 331 | 1 | Taicang | 3 | 0.01 |
| Anseong | 72 | 0.22 | Dammam | 49 | 0.15 | Iztapalapa, Mexico City | 17 | 0.05 | Nantong | 179 | 0.54 | Tangerang | 3 | 0.01 |
| Apodaca | 32 | 0.1 | Delhi | 5 | 0.02 | Jakarta | 27 | 0.08 | New Delhi | 97 | 0.29 | Tangshan | 53 | 0.16 |
| Arteaga | 4 | 0.01 | Dhaka | 10 | 0.03 | Jeddah | 84 | 0.25 | Ningbo | 714 | 2.16 | Thane | 9 | 0.03 |
| Asuncion | 3 | 0.01 | Doha | 22 | 0.07 | Jian | 29 | 0.09 | Ningde | 12 | 0.04 | Tianjin | 567 | 1.71 |
| Aurangabad | 7 | 0.02 | Dongguan | 1,070 | 3.23 | Jiangmen | 118 | 0.36 | Noida | 11 | 0.03 | Tijuana | 47 | 0.14 |
| Azcapotzalco | 15 | 0.05 | Dubai | 220 | 0.66 | Jiaxing | 190 | 0.57 | Palmira | 3 | 0.01 | Tlajomulco De Zuniga | 9 | 0.03 |
| Bac Ninh | 473 | 1.43 | Durban | 11 | 0.03 | Jinan | 107 | 0.32 | Pasig | 2 | 0.01 | Tlaxcala | 2 | 0.01 |
| Bangalore | 49 | 0.15 | El Marques | 11 | 0.03 | Johannesburg | 57 | 0.17 | Penang | 7 | 0.02 | Toluca | 9 | 0.03 |
| Baoding | 61 | 0.18 | Escobedo | 23 | 0.07 | Johor Bahru | 16 | 0.05 | Petaling Jaya | 10 | 0.03 | Torreon | 4 | 0.01 |
| Barranquilla | 7 | 0.02 | Faridabad | 8 | 0.02 | Juarez | 2 | 0.01 | Pretoria | 5 | 0.02 | Uitenhage | 2 | 0.01 |
| Beijing | 608 | 1.84 | Foshan | 404 | 1.22 | Jundiai | 77 | 0.23 | Puebla | 68 | 0.21 | Vadodara | 13 | 0.04 |
| Betim | 77 | 0.23 | Funza | 2 | 0.01 | Kancheepuram | 4 | 0.01 | Pune | 49 | 0.15 | Visakhapatnam | 2 | 0.01 |
| Bhiwadi | 2 | 0.01 | Fuzhou | 152 | 0.46 | Karachi | 10 | 0.03 | Pyeongtaek | 211 | 0.64 | Wan Chai | 18 | 0.05 |
| Bogota | 252 | 0.76 | Gandhinagar | 3 | 0.01 | Kochi | 2 | 0.01 | Qingdao | 259 | 0.78 | Wuhan | 356 | 1.07 |
| Buenos Aires | 4 | 0.01 | Ghaziabad | 2 | 0.01 | Kolkata | 77 | 0.23 | Queretaro | 73 | 0.22 | Wuxi | 658 | 1.99 |
| Bursa | 4 | 0.01 | Gimpo-si | 293 | 0.88 | Kuala Lumpur | 24 | 0.07 | Quito | 9 | 0.03 | Xi'an | 31 | 0.09 |
| Busan | 1,114 | 3.36 | Gravatai | 58 | 0.18 | Kuching | 2 | 0.01 | Ramos Arizpe | 8 | 0.02 | Xian | 46 | 0.14 |
| Cairo | 149 | 0.45 | Guadalajara | 56 | 0.17 | Kunming | 14 | 0.04 | Resende | 14 | 0.04 | Xiangyang | 30 | 0.09 |
| Calama | 17 | 0.05 | Guangzhou | 597 | 1.8 | Kunshan | 16 | 0.05 | Rio De Janeiro | 21 | 0.06 | Xiaogan | 6 | 0.02 |
| Campinas | 3 | 0.01 | Guiyang | 11 | 0.03 | Kuwait City | 10 | 0.03 | Riyadh | 130 | 0.39 | Xingtai | 22 | 0.07 |
| Can Tho | 5 | 0.02 | Gurgaon | 15 | 0.05 | Lagos | 5 | 0.02 | SAO Paulo | 4 | 0.01 | Yancheng | 55 | 0.17 |
| Cape Town | 15 | 0.05 | Ha Noi | 2,022 | 6.1 | Lahore | 13 | 0.04 | Saltillo | 11 | 0.03 | Yantai | 127 | 0.38 |
| Celaya | 8 | 0.02 | Hai Duong | 10 | 0.03 | Langfang | 76 | 0.23 | San Juan Del Rio | 5 | 0.02 | Yichun | 12 | 0.04 |
| Chandigarh | 6 | 0.02 | Hai Phong | 771 | 2.33 | Leon | 19 | 0.06 | San Luis Potosi | 93 | 0.28 | Yongin | 175 | 0.53 |
| Changchun | 107 | 0.32 | Hanam | 16 | 0.05 | Lerma | 11 | 0.03 | Santiago | 61 | 0.18 | Yulin | 3 | 0.01 |
| Changsha | 129 | 0.39 | Hangzhou | 514 | 1.55 | Lima | 8 | 0.02 | Sao Paulo | 2,262 | 6.83 | Zapopan | 52 | 0.16 |
| Changshu | 13 | 0.04 | Haryana | 2 | 0.01 | Linyi | 23 | 0.07 | Seongnam | 229 | 0.69 | Zhangjiagang | 19 | 0.06 |
| Changwon | 524 | 1.58 | Hebi | 5 | 0.02 | Luoyang | 49 | 0.15 | Seoul | 1,276 | 3.85 | Zhengzhou | 95 | 0.29 |
| Changzhou | 424 | 1.28 | Hefei | 203 | 0.61 | Makati | 3 | 0.01 | Shah Alam | 2 | 0.01 | Zhenjiang | 121 | 0.37 |
| Chengdu | 244 | 0.74 | Heyuan | 14 | 0.04 | Manama (Al-Manamah) | 7 | 0.02 | Shanghai | 1,788 | 5.4 | Zhuhai | 210 | 0.63 |
| Chennai | 59 | 0.18 | Ho Chi Minh | 2,915 | 8.8 | Manaus | 16 | 0.05 | Shaoxing | 45 | 0.14 |  |  |  |
| Cheongju-SI | 109 | 0.33 | Hohhot | 2 | 0.01 | Medellin | 2 | 0.01 | Sharjah | 15 | 0.05 |  |  |  |
| Cheung Sha Wan | 16 | 0.05 | Hong Kong | 7 | 0.02 | Mexicali | 2 | 0.01 | Shenyang | 180 | 0.54 |  |  |  |
| Chihuahua | 15 | 0.05 | Huai’An | 4 | 0.01 | Mexico City | 87 | 0.26 | Shenzhen | 2,111 | 6.37 | Total | 33,126 | 100 |

Table A.5. Correlation matrix

First stage

|  |  |  | 1 | 2 | 3 | 4 | 5 | 6 | 7 | 8 | 9 | 10 |
| --- | --- | --- | --- | --- | --- | --- | --- | --- | --- | --- | --- | --- |
| 1 | DV: labour productivity | | 1 |  |  |  |  |  |  |  |  |  |
| 2 | FDI project value | | 0.0040 | 1 |  |  |  |  |  |  |  |  |
| 3 | Capital value other FDI projects | | 0.0686 | 0.0117 | 1 |  |  |  |  |  |  |  |
| 4 | Share of other FDI projects | | 0.0773 | -0.3543 | 0.7997 | 1 |  |  |  |  |  |  |
| 5 | Firm age |  | 0.1795 | -0.034 | 0.0551 | 0.1044 | 1 |  |  |  |  |  |
| 6 | Firm size: Large | | 0.0320 | 0.0231 | 0.0934 | 0.086 | 0.2570 | 1 |  |  |  |  |
| 7 | Firm size: Small | | 0.0519 | -0.0297 | -0.1996 | -0.1994 | -0.2821 | -0.4574 | 1 |  |  |  |
| 8 | No. firms in sector-city | | -0.0552 | -0.0194 | 0.2894 | 0.2546 | -0.0211 | 0.0269 | -0.1012 | 1 |  |  |
| 9 | No. firms in other sectors-city | | 0.0332 | -0.0155 | 0.4107 | 0.3699 | 0.0113 | 0.0788 | -0.1839 | 0.6407 | 1 |  |
| 10 | Tot. assets in city-sector | | 0.0595 | 0.0067 | 0.2310 | 0.2127 | 0.0395 | 0.1231 | -0.1697 | 0.3680 | 0.3666 | 1 |

Second stage

|  |  | 1 | 2 | 3 | 4 | 5 | 6 | 7 | 8 | 9 | 10 | 11 | 12 | 13 |
| --- | --- | --- | --- | --- | --- | --- | --- | --- | --- | --- | --- | --- | --- | --- |
| 1 | Rule of Law | 1 |  |  |  |  |  |  |  |  |  |  |  |  |
| 2 | Control of corruption | 0.9531 | 1 |  |  |  |  |  |  |  |  |  |  |  |
| 3 | Property rights | 0.8015 | 0.8785 | 1 |  |  |  |  |  |  |  |  |  |  |
| 4 | Aggreg. Labprod in the host city | -0.0186 | -0.0068 | 0.0024 | 1 |  |  |  |  |  |  |  |  |  |
| 5 | No. firms in sector-city | -0.2073 | -0.1209 | -0.0418 | 0.2737 | 1 |  |  |  |  |  |  |  |  |
| 6 | No. firms in other sectors-city | -0.2842 | -0.1952 | -0.1521 | 0.315 | 0.8854 | 1 |  |  |  |  |  |  |  |
| 7 | GDP | -0.5868 | -0.5187 | -0.3964 | 0.1843 | 0.6186 | 0.6024 | 1 |  |  |  |  |  |  |
| 8 | GDP per capita | 0.6952 | 0.7755 | 0.7698 | 0.0294 | -0.0236 | -0.0778 | -0.3012 | 1 |  |  |  |  |  |
| 9 | Unemployment rate | -0.1259 | -0.1669 | -0.0307 | -0.0054 | -0.1557 | -0.137 | -0.034 | -0.203 | 1 |  |  |  |  |
| 10 | Inflation, GDP deflator | -0.1812 | -0.2814 | -0.2995 | -0.0292 | -0.1748 | -0.1811 | 0.0175 | -0.2895 | 0.3084 | 1 |  |  |  |
| 11 | IFDI | -0.2269 | -0.1252 | -0.0032 | 0.0916 | 0.5613 | 0.4614 | 0.7984 | -0.0153 | -0.1715 | -0.0465 | 1 |  |  |
| 12 | Geo distance (capital to capital) | 0.2463 | 0.2319 | 0.2178 | -0.027 | -0.0024 | -0.0495 | -0.1095 | 0.1393 | 0.0328 | -0.0298 | -0.0097 | 1 |  |
| 13 | Type: Greenfield | 0.0148 | 0.0263 | 0.0387 | -0.0451 | -0.0569 | -0.0349 | -0.0888 | 0.0482 | -0.0118 | -0.0115 | -0.0632 | 0.2224 | 1 |

Figure A.1. Kernel density distribution of the estimated FDI project‐level parameter of FDI productivity spillovers (FDI project value variable – stage one).

Source: Authors' elaboration from estimation presented in Table 2
